# Update and nomenclature proposal for mammalian lysophospholipid acyltransferases, which create membrane phospholipid diversity

**DOI:** 10.1016/j.jbc.2021.101470

**Published:** 2021-12-07

**Authors:** William J. Valentine, Keisuke Yanagida, Hiroki Kawana, Nozomu Kono, Nobuo N. Noda, Junken Aoki, Hideo Shindou

**Affiliations:** 1 Department of Lipid Signaling, National Center for Global Health and Medicine (NCGM), Shinjuku-ku, Tokyo, Japan; 2 Department of Molecular Therapy, National Institute of Neuroscience, National Center of Neurology and Psychiatry (NCNP), Kodaira, Tokyo, Japan; 3 Department of Health Chemistry, Graduate School of Pharmaceutical Sciences, The University of Tokyo, Bunkyo-ku, Tokyo, Japan; 4 Institute of Microbial Chemistry (BIKAKEN), Microbial Chemistry Research Foundation, Shinagawa-ku, Tokyo, Japan; 5 Department of Lipid Medical Science, Graduate School of Medicine, The University of Tokyo, Bunkyo-ku, Tokyo, Japan

**Keywords:** LPLAT, glycerophospholipid, enzyme nomenclature, cellular membrane, AGPAT, 1-acylglycerol-3-phosphate *O*-acyltransferase, BAT, brown adipose tissue, CDP-DAG, cytidine diphosphate-DAG, CGL, congenital generalized lipodystrophy, CHP1, calcineurin B homologous protein 1, CL, cardiolipin, COX, cyclooxygenase, DAG, diacylglycerol, DGAT, DAG acyltransferase, DHA, docosahexaenoic acid, ER, endoplasmic reticulum, G3P, glycerol-3-phosphate, GNPAT, glyceronephosphate *O*-acyltransferase, GOAT, ghrelin *O*-acyltransferase, GPAT, G3P acyltransferase, HHAT, hedgehog acyl-transferase, KO, knockout, LCL, lyso-CL, LCLAT, LCL acyltransferase, LPA, lyso-PA, LPAAT, LPA acyltransferase, LPC, lyso-PC, LPCAT, LPC acyltransferase, LPE, lyso-PE, LPEAT, LPE acyltransferase, LPG, lyso-PG, LPGAT, LPG acyltransferase, LPI, lyso-PI, LPIAT, LPI acyltransferase, LPL, lysophospholipid, LPLAT, LPL acyltransferase, LPS, lyso-PS, LPSAT, LPS acyltransferase, MBOAT, membrane bound *O*-acyltransferase, NCBI, National Center for Biotechnology Information, NEM, N-ethylmaleimide, PA, phosphatidic acid, PAF, platelet-activating factor, PAFR, PAF receptor, PAP, PA phosphatase, PC, phosphatidylcholine, PE, phosphatidylethanolamine, PG, phosphatidylglycerol, PI, phosphatidylinositol, PL, phospholipid, PLA, phospholipase A, pLDDT, predicted Local Distance Difference Test, PORCN, porcupine *O*-acyltransferase, PS, phosphatidylserine, *sn*, stereospecifically numbered, SOAT, sterol *O*-acyltransferase, srebp, sterol regulatory element binding protein, TAG, triacylglycerol, WAT, white adipose tissue

## Abstract

The diversity of glycerophospholipid species in cellular membranes is immense and affects various biological functions. Glycerol-3-phosphate acyltransferases (GPATs) and lysophospholipid acyltransferases (LPLATs), in concert with phospholipase A_1/2_s enzymes, contribute to this diversity *via* selective esterification of fatty acyl chains at the *sn*-1 or *sn*-2 positions of membrane phospholipids. These enzymes are conserved across all kingdoms, and in mammals four GPATs of the 1-acylglycerol-3-phosphate *O*-acyltransferase (AGPAT) family and at least 14 LPLATs, either of the AGPAT or the membrane-bound *O*-acyltransferase (MBOAT) families, have been identified. Here we provide an overview of the biochemical and biological activities of these mammalian enzymes, including their predicted structures, involvements in human diseases, and essential physiological roles as revealed by gene-deficient mice. Recently, the nomenclature used to refer to these enzymes has generated some confusion due to the use of multiple names to refer to the same enzyme and instances of the same name being used to refer to completely different enzymes. Thus, this review proposes a more uniform LPLAT enzyme nomenclature, as well as providing an update of recent advances made in the study of LPLATs, continuing from our JBC mini review in 2009.

## Diversity of cellular membrane glycerophospholipids

Biological membranes of mammalian cells are comprised mostly of proteins and lipids. Glycerophospholipids (phospholipids), along with sphingolipids and cholesterol, are the major lipid components. The basic structure of membrane phospholipids, with a glycerol backbone, polar headgroup, and two hydrophobic chains, provides a template for a diverse array of chemical species to support their many biological functions. These functions include formation of lipid bilayers that form hydrophobic barriers in cellular membranes, which encapsulate and compartmentalize cells as well as provide domains of molecular interactions (1). Phospholipids also form monolayers, which encapsulate lipid and lipoprotein particles or form tissue surface films such as pulmonary surfactant (2, 3). Phospholipids of biological membranes also provide precursor molecules for lipid mediators involved in inflammation and resolution (4, 5). To perform these diverse functions, a wide variety of phospholipid species exists, and their compositions in cellular membranes vary among organelles, cell types, and tissues to impart the membranes with essential properties for their biological functions (1, 6).

**Figure 1:**
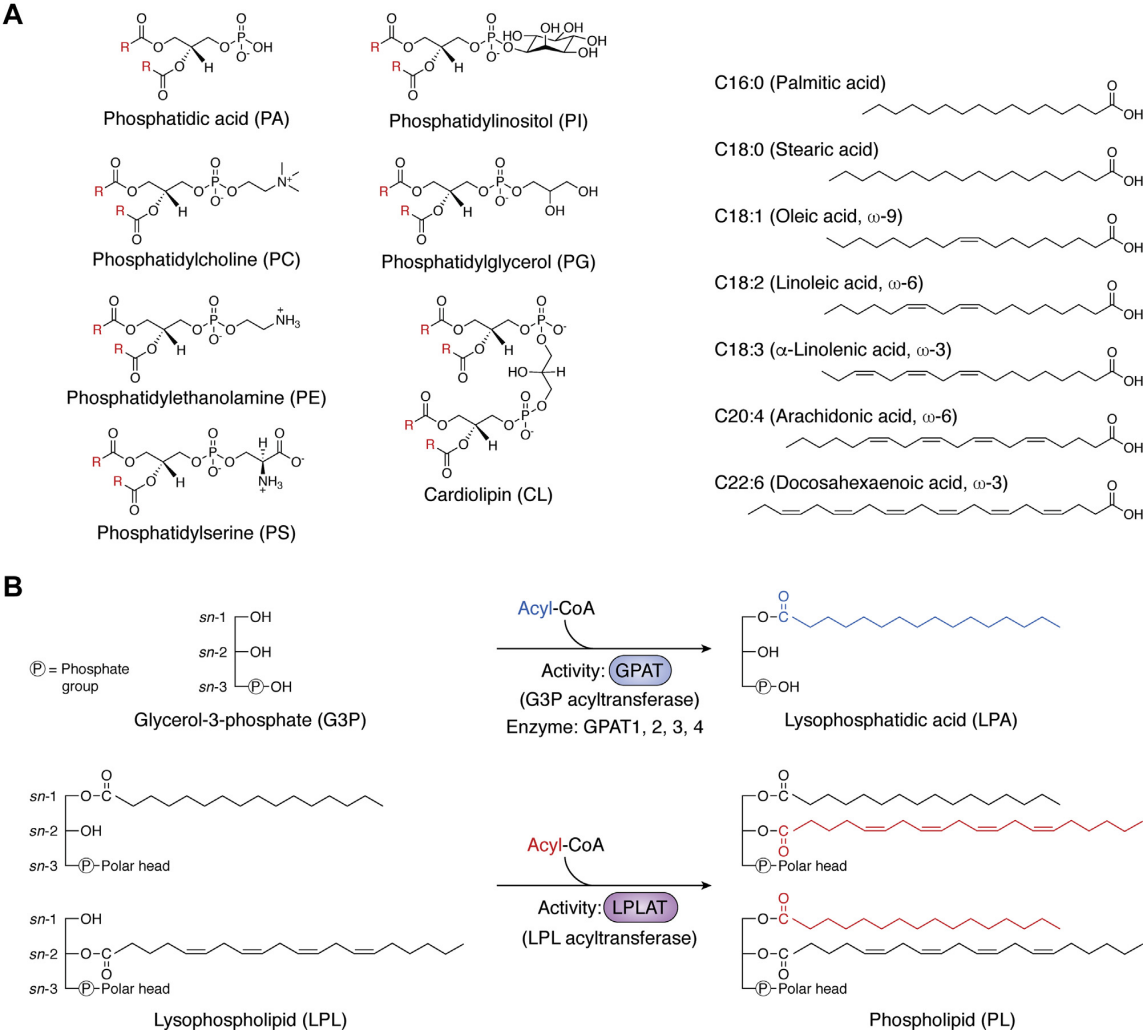
**Chemical structures of mammalian phospholipids and two types of acyltransferases to biosynthesize phospholipids.***A*, in the *left panel*, structures of the major classes of mammalian phospholipids are shown. "R" (highlighted in *red*) indicates hydrocarbon chains of fatty acids. In the *right panel*, several of the fatty acids most commonly esterified in mammalian phospholipids are shown. *B*, *upper*, GPATs synthesize LPA using G3P and acyl-CoA as substrates. Currently four GPATs, GPAT1 to 4, have been identified, all of the AGPAT family. *B*, *lower*, LPLATs biosynthesize phospholipids using *sn*-1- or *sn*-2-acyl LPLs and acyl-CoAs as substrates. The LPLs may be of several classes, including LPA. Currently, 14 LPLATs have been identified, all either from the AGPAT or MBOAT families.

Chemical diversity of phospholipids is introduced largely by the choices of headgroups at the stereospecifically numbered (*sn*) -3 position and two fatty acids and linkages at the *sn*-1 and *sn*-2 positions of their glycerol-based structures (1) (Fig. 1*A*). For headgroups, the simplest phospholipid class, phosphatidic acid (PA), has just a phosphate. During *de novo* phospholipid synthesis, PA is a precursor molecule for the production of other phospholipids with more complex headgroups: phosphatidylcholine (PC), phosphatidylethanolamine (PE), phosphatidylserine (PS), phosphatidylinositol (PI), phosphatidylglycerol (PG), and cardiolipin (CL) (7, 8). Chemical diversity is also generated from the choices of fatty chains introduced at *sn*-1 and *sn*-2. Carbon chain lengths and double-bond numbers vary, usually between 12 and 24 carbons and between 0 and 6 double bonds, and the linkages of the chains to the glycerol backbone also vary. Acyl linkages are most common at both *sn*-1 and *sn*-2; however, alkyl and alkenyl linkages also occur at *sn*-1 to form plasmanyl- and plasmenyl-phospholipids, respectively (9). The membrane phospholipid diversity generated by the combinations of headgroups, fatty chains, and linkages is extensive and comprised of over a thousand species (7, 10).

The choices of headgroups, fatty acids, and linkages are all essential features that affect phospholipid function. For the fatty acids, carbon chain lengths and double-bond numbers/positions are important attributes, which may affect biophysical and biological properties of phospholipid layers (11, 12). These include biomembrane fluidity, flexibility, thickness, and curvature (13, 14), as well as propensity to form signaling domains or provide specific polyunsaturated chains for conversion to lipid mediators (4, 11). The diversity of fatty chain sets is determined in two distinct steps of *de novo* phospholipid biosynthesis and phospholipid remodeling, as described in detail in Phospholipid biosynthesis section.

## Phospholipid biosynthesis

**Figure 2:**
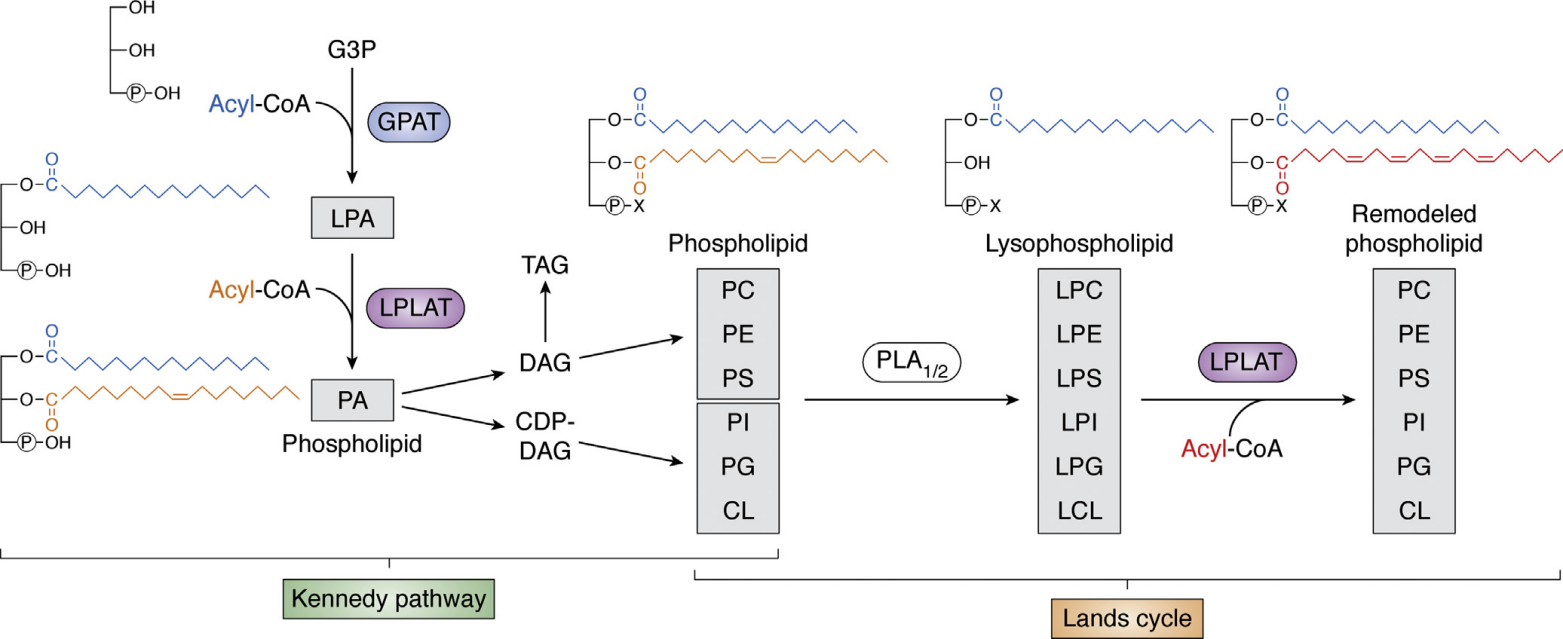
**Biosynthetic pathways of phospholipid diversity.** Phospholipids are first produced in the Kennedy pathway by acylation of G3P by GPAT to produce LPA, and subsequent acylation by LPLAT (LPAAT reaction in this case) to produce PA. This PA may be further metabolized to DAG and used to produce PC, PE, and PS, as well as TAG. Alternatively, the PA may be used to produce CDP-DAG and further metabolized to PI, PG, and CL. The phospholipids produced in the Kennedy pathway are remodeled by the concerted action of PLA_1/2_ and LPLAT in the Lands cycle. Lands cycle remodeling of a *sn*-2-acyl chain is illustrated; however, remodeling of *sn*-1 may also occur. The representative fatty acids shown are palmitic acid (*blue*), oleic acid (*orange*), and arachidonic acid (*red*). X represents any phospholipid polar head group. CDP-DAG, cytidine diphosphate-DAG; CL, cardiolipin; DAG, diacylglycerol; G3P, glycerol-3-phosphate; GPAT, G3P acyltransferase; LPL, lysophospholipid; LPLAT, LPL acyltransferase; PA, phosphatidic acid; PC, phosphatidylcholine; PE, phosphatidylethanolamine; PG, phosphatidylglycerol; PI, phosphatidylinositol; PLA, phospholipase; PS phosphatidylserine; TAG, triacylglycerol.

Phospholipids can be produced by several metabolic routes, and a key reaction that utilizes lysophospholipids and acyl-CoAs as substrates to produce phospholipids is catalyzed by a class of enzymes called lysophospholipid acyltransferases (LPLATs) (Fig. 1*B*). Chemical diversity is endowed to phospholipid molecules in two distinct phases of their biosynthesis, the Kennedy pathway (*de novo* pathway) (15) and the Lands cycle (remodeling pathway) (16) (Fig. 2). These pathways were both first proposed in the 1950s, and multiple different LPLATs function in both pathways (17).

In the Kennedy pathway, where phospholipids are newly synthesized, glycerol-3-phosphate (G3P) acyltransferases (GPATs) use G3P and acyl-CoA as substrates to produce a key intermediate in phospholipid synthesis, lysophosphatidic acid (LPA) (Figs. 1*B* and 2). This LPA is converted to phosphatidic acid (PA) through the introduction of a second fatty acid in a LPLAT-catalyzed reaction. This type of LPLAT reaction that produces PA may also be referred to as an LPA acyltransferase (LPAAT) reaction (7, 18). The PA they produce may be further converted to the other classes of phospholipids *via* two main routes. In one route, PA is dephosphorylated by PA phosphatases (PAPs; also known as lipins) to produce diacylglycerol (DAG). DAG may be next metabolized to PC and PE, which may be further converted to PS by PS synthases (19). DAG may also be utilized as substrate by DAG acyltransferases (DGATs) to produce triacylglycerol (TAG) (20). In the other route, PA is changed to cytidine diphosphate (CDP)-DAG by CDP-DAG synthase and further metabolized to produce PI, PG, and CL. Through these reactions, phospholipids having a variety of chain sets and all types of polar head groups are newly biosynthesized during the Kennedy pathway (Fig. 2).

Cellular phospholipids synthesized in the Kennedy pathway are further subjected to fatty chain remodeling reactions in the pathway known as the Lands cycle. In this cycle, fatty acids of phospholipids are selectively replaced by the concerted actions of phospholipase (PLA)_1_/_2_s and LPLATs (7, 21) (Figs. 1*B* and 2). Cells can also take up phospholipids such as by endocytosis (22, 23), and these imported phospholipids may undergo Lands cycle remodeling. Multiple LPLATs with distinct substrate specificities are active in the Lands cycle, and the levels of these enzymes and the availability of their substrates are major determinants of the compositional diversity of phospholipid species in cells (14).

**Figure 3:**
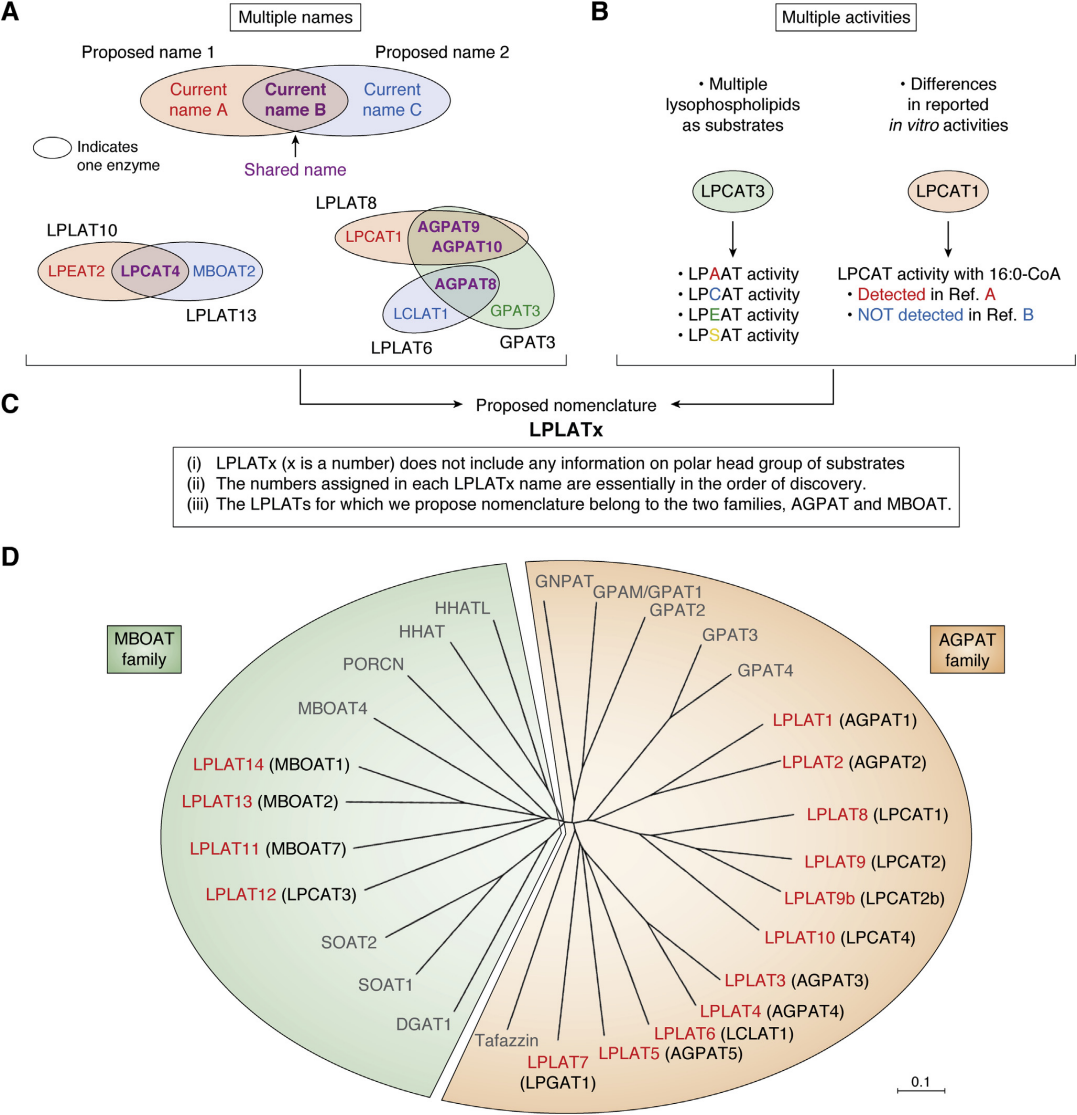
**Criteria of proposed nomenclature to overcome the current problems and dendrogram of AGPAT and MBOAT family members.***A*, multiple naming of individual enzymes in the current nomenclature. In several cases, the same name has been assigned to completely different enzymes. *Purple shading* indicates identical names shared by different enzymes, which may generate confusion. *B*, current nomenclature inaccurately reflects enzymatic activities. *B*, *left*, most LPLATs utilize multiple lysophospholipids as substrates. *B*, *right*, the reported substrate selectivities *in vitro* often differ between studies. *C*, the proposed nomenclature is based on three criteria, as described in Nomenclature section. *D*, the dendrogram was drawn using ClustalW (https://clustalw.ddbj.nig.ac.jp) and MEGA X. AGPAT family members include LPLATs, GPATs, Tafazzin (NP_001167018), and GNPAT (NP_034452). Tafazzin and GNPAT are reported to have transacylase and dihydroxyacetone phosphate activity, respectively (18, 183). MBOAT family members include LPLATs, diacylglycerol acyltransferase 1 (DGAT1, NP_034176), sterol *O*-acyltransferase 1 (SOAT1, NP_033256) and SOAT2 (NP_666176), and protein acyltransferases including MBOAT4 (also called ghrelin *O*-acyltransferase, GOAT, NP_001119786), porcupine *O*-acyltransferase (PORCN, NP_665914), hedgehog acyltransferase (HHAT, NP_659130), and HHATL (HHAT-like, NP_083371). LPLATs in both families possess motifs (either AGPAT motifs or MBOAT motifs) essential for their LPLAT activities. For LPLATs, new proposed names are shown in *red*, followed by the current official symbols in *parentheses* (in *black*).

Now 14 mammalian LPLATs are reported that function in the Kennedy pathway and/or Lands cycle (7, 17). Based on their primary structures, these LPLATs are divided into two families, the 1-acylglycerol-3-phosphate *O*-acyltransferase (AGPAT) and the membrane bound *O*-acyltransferase (MBOAT) families (Fig. 3*D*, see also Table 1) (7, 17). These two families also contain non-LPLAT members. The AGPAT family also contains GPATs and tafazzin, an enzyme with CoA-independent transacylase activity (18). The MBOAT family also contains DGAT1, sterol *O*-acyltransferase (SOAT) 1 and 2 (also called acyl-CoA:cholesterol acyltransferase 1 and 2), and several protein acyltransferases such as ghrelin *O*-acyltransferase (GOAT), porcupine *O*-acyltransferase (PORCN), hedgehog acyltransferase (HHAT), and HHAT-like (24). Thus, different members of the two families, AGPAT and MBOAT, possess primary activities to acylate not just lysophospholipids but also other protein and lipid substrates. The diversity of acylating activities among these enzymes is immense, and in some cases the specific primary biological activities of the enzymes in terms of specific substrates utilized and products generated are still not clear. As discussed in Nomenclature section, this also applies to some of the 14 enzymes functionally classified as LPLATs.

**Table 1:** Proposed nomenclature of LPLATs

Family	Proposed name	NCBI				
		Current official symbol	Organism	Reference sequence	Gene ID	Also known as
AGPAT	LPLAT1	AGPAT1	Human	NM_032741	10554	G15; LPAATA; 1-AGPAT1; LPAAT-alpha
		Agpat1	Mouse	NM_018862	55979	1-A; Lpa; 1-AGP; 1-AGPAT; AW047140
	LPLAT2	AGPAT2	Human	NM_006412	10555	BSCL; BSCL1; LPAAB; 1-AGPAT2; LPAAT-beta
		Agpat2	Mouse	NM_026212	67512	BSC; BSCL; BSCL1; LPAAB; LPAAT; AV000834; LPAAT-beta; 2510002J07Rik
	LPLAT3	AGPAT3	Human	NM_001037553	56894	LPAAT3; 1-AGPAT 3; LPAAT-GAMMA1
		Agpat3	Mouse	NM_053014	28169	LP; lpaat3; AW061257; AW493985; D10Jhu12e
	LPLAT4	AGPAT4	Human	NM_020133	56895	1-AGPAT4; dJ473J16.2; LPAAT-delta
		Agpat4	Mouse	NM_026644	68262	1500003P24Rik
	LPLAT5	AGPAT5	Human	NM_018361	55326	LPAATE; 1AGPAT5
		Agpat5	Mouse	NM_026792	52123	D8Ertd319; D8Ertd319e; 1110013A05Rik
	LPLAT6	LCLAT1	Human	NM_182551	253558	LYCAT; AGPAT8; ALCAT1; 1AGPAT8; UNQ1849; HSRG1849
		Lclat1	Mouse	NM_001177968	225010	AGP; ALC; Lyc; Gm91; Lycat; Agpat8; Alcat1; AI181996; 1-AGPAT 8
	LPLAT7	LPGAT1	Human	NM_014873	9926	NET8; FAM34A; FAM34A1
		Lpgat1	Mouse	NM_172266	226856	AI649174; AW112037; BC013667
	LPLAT8	LPCAT1	Human	NM_024830	79888	AYTL2; lpcat; AGPAT9; PFAAP3; AGPAT10; LPCAT-1; lysoPAFAT
		Lpcat1	Mouse	NM_145376	210992	LP; Ayt; rd1; rd11; Aytl2; LPCAT; C87117; LPCAT-1; mLPCAT1; BB137372; BC005662; lysoPAFAT; 2900035H07Rik
	LPLAT9	LPCAT2	Human	NM_017839	54947	AYTL1; AGPAT11; LysoPAFAT
		Lpcat2	Mouse	NM_173014	270084	Ayt; LPC; Aytl1; Aytl1a; lpafat1; lysoPAFAT; 1-AGPAT 11; A330042H22; lysoPAFAT/LPCAT2
	LPLAT9b	Lpcat2b	Mouse	NM_027599	70902	Aytl; Aytl1b; 4921521K07Rik
	LPLAT10	LPCAT4	Human	NM_153613	254531	AYTL3; AGPAT7; LPEAT2; LPAAT-eta
		Lpcat4	Mouse	NM_207206	99010	Agp; Ayt; LPE; Aytl3; Agpat7; LPEAT2; AI505034
MBOAT	LPLAT11	MBOAT7	Human	NM_024298	79143	BB1; LRC4; LENG4; LPIAT; LPLAT; MBOA7; MRT57; OACT7; hMBOA-7
		Mboat7	Mouse	NM_029934	77582	Lp; BB1; Len; Leng4; Lpiat; Lpiat1; LPLAT 7; m-mboa-7; 5730589L02Rik
	LPLAT12	LPCAT3	Human	NM_005768	10162	C3F; LPCAT; LPSAT; OACT5; nessy; MBOAT5; LPLAT 5
		Lpcat3	Mouse	NM_145130	14792	C3f; Oac; PTG; Mboa; Grcc3; Lpcat; Lpeat; Lpsat; Oact5; Grcc3f; Lplat5; Mboat5; Moact5
	LPLAT13	MBOAT2	Human	NM_138799	129642	LPAAT; LPEAT; OACT2; LPCAT4; LPLAT 2
		Mboat2	Mouse	NM_026037	67216	Oac; Oact2; LPCAT4; Moact2; AU022889; AW547221; 2810049G06Rik
	LPLAT14	MBOAT1	Human	NM_001080480	154141	LPLAT; LPSAT; OACT1; LPEAT1; LPLAT 1; dJ434O11.1
		Mboat1	Mouse	NM_153546	218121	Oac; Oact1; LPEAT1; Moact1; BC023845; 9130215M02Rik

## Nomenclature

Because LPLATs are membrane-associated enzymes and utilize lipid substrates, their purification and identification had long been elusive after the discoveries of the Kennedy pathway and the Lands cycle in 1950s. However, several LPLATs were identified that functioned in either the Kennedy pathway or the Lands cycle in the 1990s and the 2000s, respectively; 40 to 50 years after the first discoveries of both pathways. Following completion of the human genome project in 2003, multiple additional putative LPLATs were identified in the DNA databases, and consequently their characterizations as LPLATs were performed in rapid succession, especially around 2004 to 2009 (7). As a result, currently a total of 14 LPLATs from the AGPAT (ten LPLATs) and MBOAT (four LPLATs) families are now known, and they are each conserved between human and mouse. In the course of these studies, many enzymes were assigned different names by different groups, and also registered in genomic databases with incorrect information of their substrate specificities as LPLATs, resulting in multiple names being assigned to each LPLAT enzyme. In several instances, the same name was assigned to completely different enzymes, which has generated some confusion in the literature and research field in referring to specific LPLATs and GPATs (Fig. 3*A*, Tables 1 and 2). For example, the name “lyso-PC acyltransferase (LPCAT)4” sometimes indicates either of two enzymes, MBOAT2 (Gene ID: 129642) or lyso-PE acyltransferase (LPEAT)2 (Gene ID: 254531). Another example is “AGPAT8,” which may indicate two enzymes, lyso-CL acyltransferase (LCLAT)1 (Gene ID: 253558) and GPAT3 (Gene ID: 84803). Similarly, LPCAT1 (Gene ID: 79888) and GPAT3 have each been referred to as both “AGPAT9” and “AGPAT10.”

**Table 2:** Current nomenclature of GPATs

Family	Name	NCBI				
		Official symbol	Organism	Reference sequence	Gene ID	Also known as
AGPAT	GPAT1	GPAM	human	NM_001244949	57678	GPAT; GPAT1
		Gpam	mouse	NM_008149	14732	GPA; P90; GPAT; GPAT1; GPAT-1
	GPAT2	GPAT2	human	NM_207328	150763	CT123
		Gpat2	mouse	NM_001081089	215456	Gpa; Gm116; xGPAT1; A530057A03Rik
	GPAT3	GPAT3	human	NM_001256421	84803	MAG1; AGPAT8; AGPAT9; AGPAT10; AGPAT 10; HMFN0839; LPAAT-theta
		Gpat3	mouse	NM_172715	231510	Agp; Agpat9; GPAT-3; mGPAT3; 1-AGPAT; AGPAT 10; 1-AGPAT 9; 4933408F15; 4933407I02Rik; A230097K15Rik
	GPAT4	GPAT4	human	NM_178819	137964	AGPAT6; LPAATZ; TSARG7; 1-AGPAT 6; LPAAT-zeta
		Gpat4	mouse	NM_018743	102247	Agp; Agpat6; Tsarg7; AU041707; AW545732

**Figure 4:**
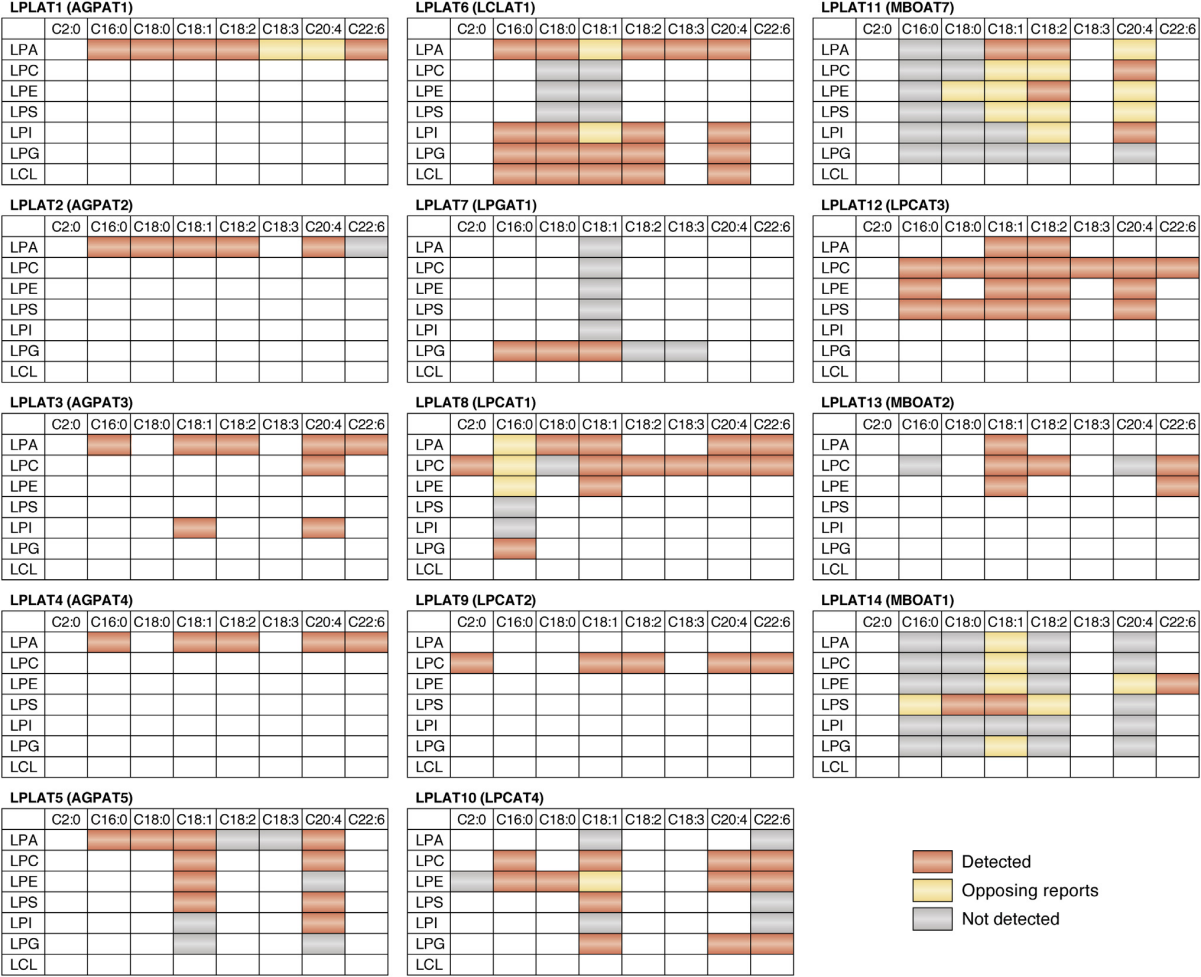
***In vitro* activities of 14 LPLATs.** The substrate recognition indicated for each enzyme is a summary based upon *in vitro* enzymatic activities reported in the literature. For each combination of lysophospholipid and fatty acyl-CoA substrates, *red* indicates the acyltransferase activity of the enzyme was “detected,” *gray* indicates the activity was “not detected,” and *orange* indicates “opposing reports” with the activity reported as both “detected” and “not detected” in different studies. For each LPLAT, the new proposed name is shown, followed by the current official symbol in *parentheses*. The summarized activities are based upon review of the literature for LPLAT1 (6, 64, 67, 80, 81, 184, 185), LPLAT2 (6, 64, 67, 80, 99, 161, 185, 186, 187, 188), LPLAT3 (6, 80, 82, 94, 189), LPLAT4 (80, 88), LPLAT5 (80, 94), LPLAT6 (98, 100, 182, 187), LPLAT7 (115, 161), LPLAT8 (6, 36, 119, 121, 130, 131, 133, 161, 182, 188, 190), LPLAT9 (6, 38, 128, 130, 131, 133, 161, 190), LPLAT10 (139, 140, 190), LPLAT11 (144, 159, 191), LPLAT12 (6, 37, 99, 140, 158, 161, 182), LPLAT13 (6, 140, 158, 159), and LPLAT14 (140, 158, 159).

In addition to the ambiguity and confusion caused by the overlapping of names, these names incompletely describe biochemical characteristics of the enzymes. Most LPLATs utilize multiple lysophospholipids as substrates (Fig. 4). For instance, LPCAT3 acts not only on lyso-PC (LPC) but also lyso-PE (LPE) and lyso-PS (LPS), but it is unclear that the enzyme has specificity to utilize several different lysophospholipids from the name “LPCAT3” (Fig. 3*B*, left and Fig. 4). Another issue is that reported substrate selectivities of some LPLATs are inconsistent among different studies, as exemplified by the case of LPCAT1 (Fig. 3*B*, right and Fig. 4). Consistent use of the unique official gene symbols provided by the National Center for Biotechnology Information (NCBI) would alleviate confusion caused by multiple naming of individual enzymes; however, many of these contain incorrect information of their LPLAT enzymatic activities, especially regarding the lysophospholipid polar head selectivity. Therefore, to avoid confusion in this research field, a revised LPLAT nomenclature is urgently needed.

In this review, we would like to propose a new nomenclature of mammalian LPLATs based on three criteria (Fig. 3*C* and Table 1). (i) LPLATx (x is a number) is used as a name, which does not include any information on polar head groups of substrates, *i.e.*, type of lysophospholipid. (ii) The numbers assigned in each LPLATx name are essentially in the order of discovery. (iii) The LPLATs for which we propose nomenclature belong to two families, AGPAT and MBOAT. Although there are a few reports of enzymes with potential LPLAT activities that belong to other protein families (25, 26, 27), this review and nomenclature proposal focus only on the enzymes of these two major families. The proposed nomenclature is shown in Table 1 along with enzyme information registered in NCBI. Nomenclature for the currently known GPATs (GPAT1-4, all AGPAT-family enzymes) is generally accurate and uniformly adopted; therefore, no revision is proposed for their current nomenclature, which is shown in Table 2 along with enzyme information registered in NCBI. We will introduce the proposed LPLATx names as our working names in this review, along with official gene symbols in parentheses as needed to avoid ambiguity, beginning with the next section, which describes enzymatic motifs and structures.

## Motifs and structures

The experimentally solved structure of a mammalian LPLAT has not been reported yet; however, the X-ray structure of PlsC, an AGPAT-family LPAAT of the bacterium *Thermotoga maritima*, has been determined (28). The model supports that PlsC introduces an acyl chain at the *sn*-2 position of PA, and the proper arrangement of hydrophobic tunnels, termed “rulers,” determines the length of the fatty acid to be introduced. In addition, an N-terminus two-helix motif anchors the protein firmly to one leaflet of the membrane (28). Recently, X-ray and Cryo-EM structures of human SOAT1, DGAT1, and HHAT, all non-LPLAT members of the MBOAT family, have been determined (29, 30, 31, 32), and it is hoped the structure of an LPLAT member of the MBOAT family will be determined soon.

Conserved motifs essential for LPLAT activities are reported and are distinct between LPLATs of the AGPAT and MBOAT families (Table 3). LPLATs and GPATs of the AGPAT family have four conserved motifs that are essential for substrate recognition and enzymatic activity: Motif 1 (xHxxxxD), Motif 2 (GxxFxxR), Motif 3 (xxEGxx), and Motif 4 (xxxxPxx) (18, 33, 34, 35, 36). MBOAT family LPLATs also contain four conserved motifs, different from the AGPAT motifs that are critical for LPLAT activities: Motif A (WD), Motif B (WHGxxxGYxxxF), Motif C (YxxxxF), and Motif D (YxxxYFxxH) (37). Recently, we reported potential acyl-CoA-binding pockets of mouse LPLAT8 (LPCAT1) and LPLAT9 (LPCAT2), both AGPAT-family LPLATs, based on homology modeling with PlsC (38). In our homology models, the four AGPAT motifs surround the putative acyl-CoA-binding pocket. LPLAT8 and LPLAT9 are thought to be monotopic membrane proteins, while LPLATs in the MBOAT family are thought to possess multiple membrane spanning domains.

**Table 3:** Structural information of LPLATs

Family	Organism	Enzyme	TM	EH	Motif				Embedded residues											
					1	2	3	4	1	2	3	4	5	6	7	8	9	10	11	12
AGPAT	human	GPAT1	0	0	230–235	272–278	313–318	347–353												
	human	GPAT2	0	0	205–210	247–253	288–293	323–329												
	human	GPAT3	1	2	228–234	268–271	300–306	328–329	3–28	142–154										
	human	GPAT4	1	1	247–253	287–290	319–325	347–348	8–36											
	human	LPLAT1	1	1	104–109	143–149	176–181	203–209	6–29											
	human	LPLAT2	1	1	98–103	137–143	170–175	197–203	3–21											
	human	LPLAT3	2	2	96–101	140–146	174–179	206	305–330	335–352										
	human	LPLAT4	2	2	98–103	140–146	174–179	198	305–330	335–355										
	human	LPLAT5	2	3	93–103	137–143	171–176	206	14–26	321–340	341–362									
	human	LPLAT6	2	2	123–128	170–176	201–206	233	343–365	369–387										
	human	LPLAT7	1	1	101–106	144–150	182–187	210	344–366											
	human	LPLAT8	0	0	135–140	172–177	206–211	229–234												
	human	LPLAT9	0	0	146–151	184–189	218–223	240–246												
	mouse	LPLAT9b	0	0	142–147	180–185	214–219	236–242												
	human	LPLAT10	0	0	129–134	167–172	201–206	224–229												


Family		Enzyme	TM	EH	A	B	C	D	1	2	3	4	5	6	7	8	9	10	11	12
MBOAT	human	LPLAT11	11	11	?	355–366	409–414	426–434	4–22	29–48	49–67	73–92	99–115	191–213	225–259	342–356	362–376	397–421	428–445	
	human	LPLAT12	11	12	313–314	373–384	434–439	451–459	39–58	68–89	90–107	112–132	141–157	184–194	230–252	265–298	358–374	380–397	418–444	453–472
	human	LPLAT13	11	12	317–318	372–383	428–433	445–453	23–42	57–75	76–93	98–118	130–143	230–251	269–282	284–302	361–373	379–397	412–438	444–462
	human	LPLAT14	11	12	325–326	380–391	436–441	453–461	31–50	65–83	84–98	108–126	138–151	185–196	238–259	277–310	369–381	387–404	420–446	458–470

Conserved Motifs were predicted by using DDBJ ClustalW (http://clustalw.ddbj.nig.ac.jp/index.php?lang=ja) and previous literature (18, 33, 34, 35, 36, 37). The number of transmembrane (TM) domains and total number of embedded helices (EH), including TMs, of the AlphaFold structures modeled in flat lipid bilayer membranes were predicted using PPM 3.0 Web Server (41). The amino acid numbering is based on corresponding reference sequences of LPLATs and GPATs indicated in Tables 1 and 2, respectively.

**Figure 5:**
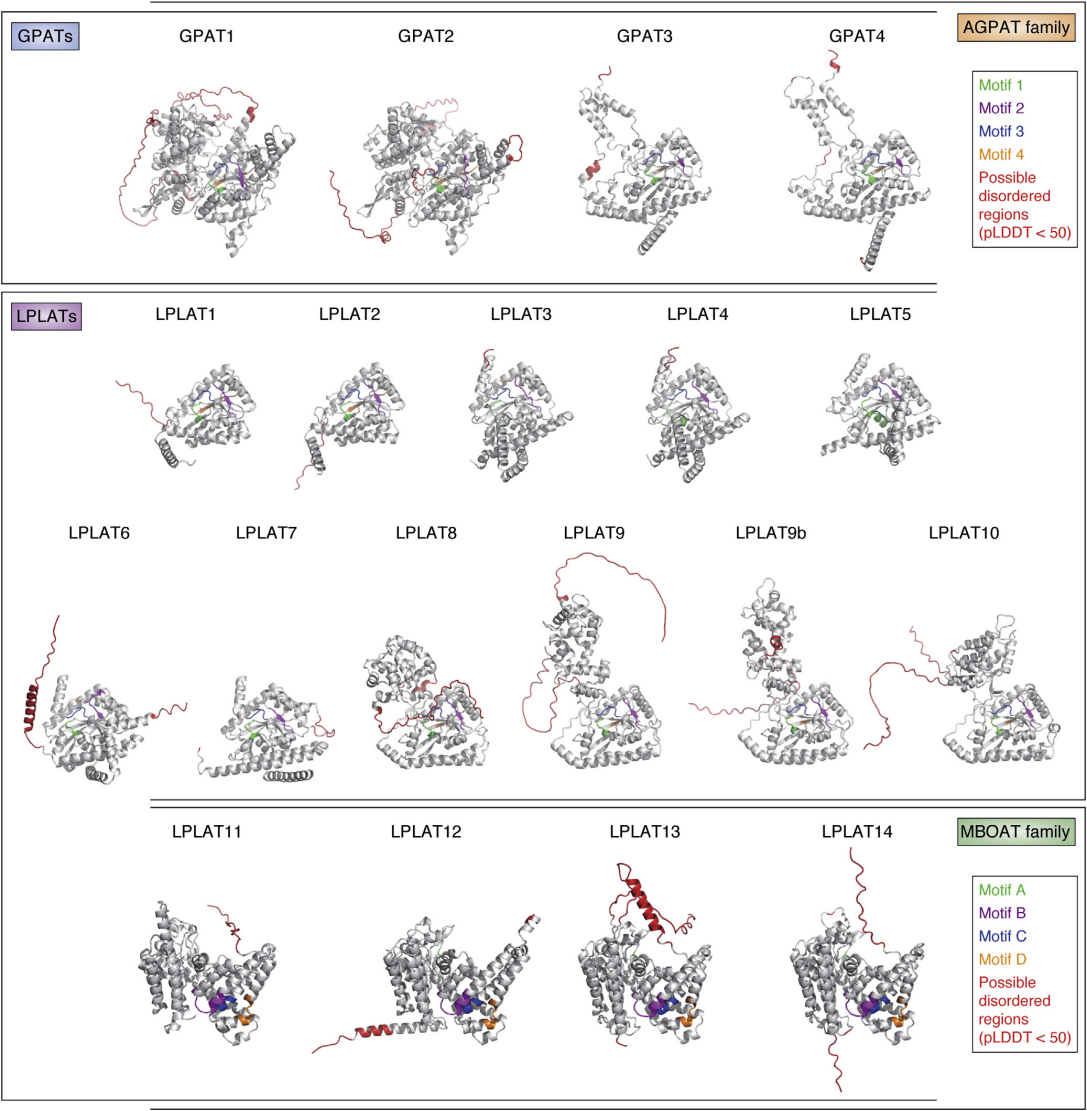
**Ribbon representation of AlphaFold structures of human GPATs and LPLATs.** All structures are of human enzymes except for mouse LPLAT9b, which has no human ortholog. AGPAT/MBOAT motifs 1/A, 2/B, 3/C, and 4/D are colored *green*, *magenta*, *blue*, and *orange*, respectively. Regions predicted with low confidence (predicted Local Distance Difference Test, pLDDT, less than 50), including inherently disordered regions, are colored *red*. The numbered amino acid residues of the AGPAT and MBOAT motifs are indicated in Table 3.

**Figure 6:**
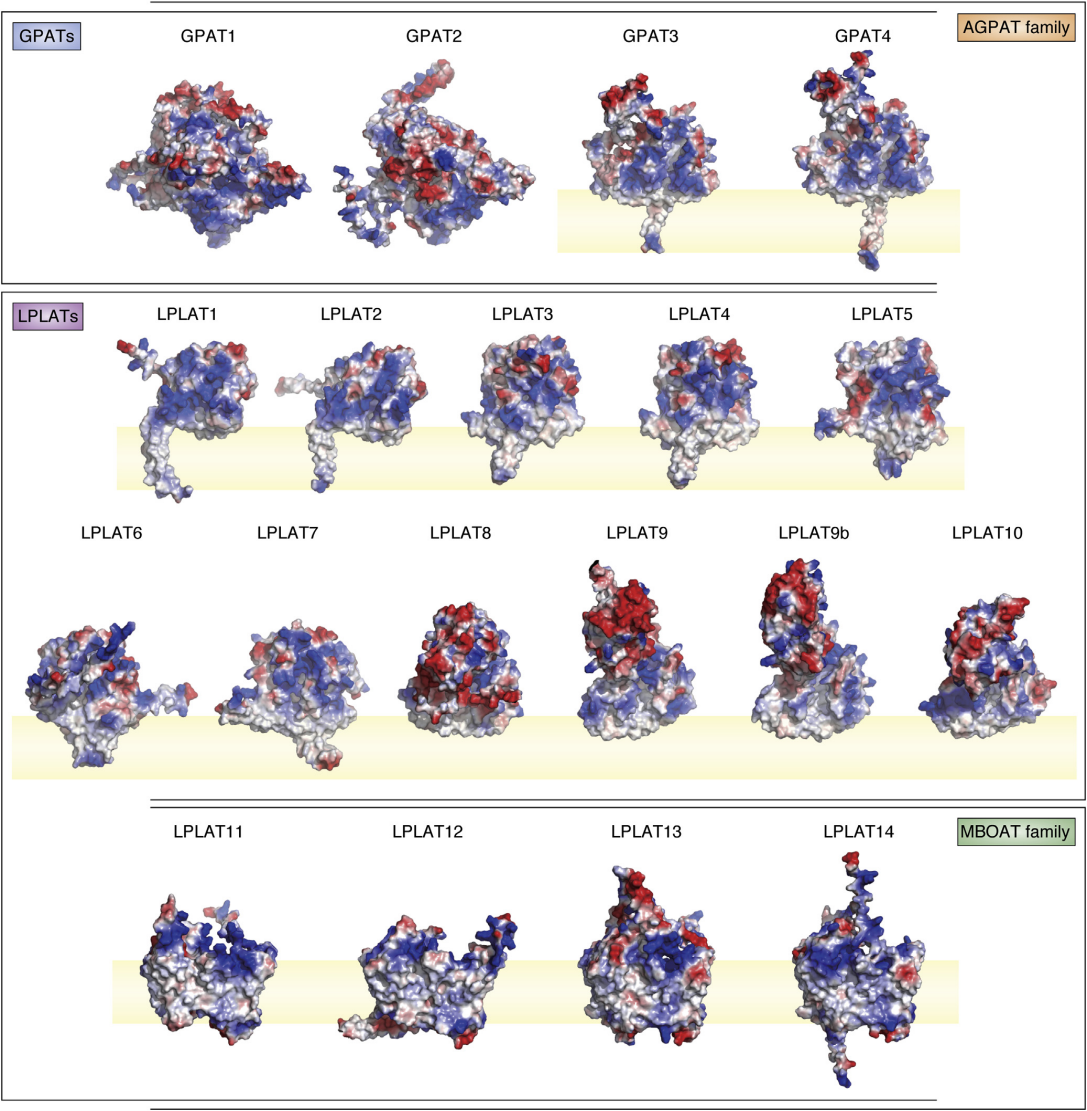
**Surface charge representations of AlphaFold structures of human GPATs and LPLATs.** All structures are of human enzymes except for mouse LPLAT9b, which has no human ortholog. Electrostatic charges were calculated using the default vacuum electrostatic package in PyMOL. *Red* and *blue shading* represents negatively and positively charged regions, respectively, whereas *white shading* represents neutral/hydrophobic regions. Protein structure regions overlapping with *light-yellow shaded areas* (representing lipid bilayer) indicate regions predicted to be embedded in the membrane.

**Figure 7:**
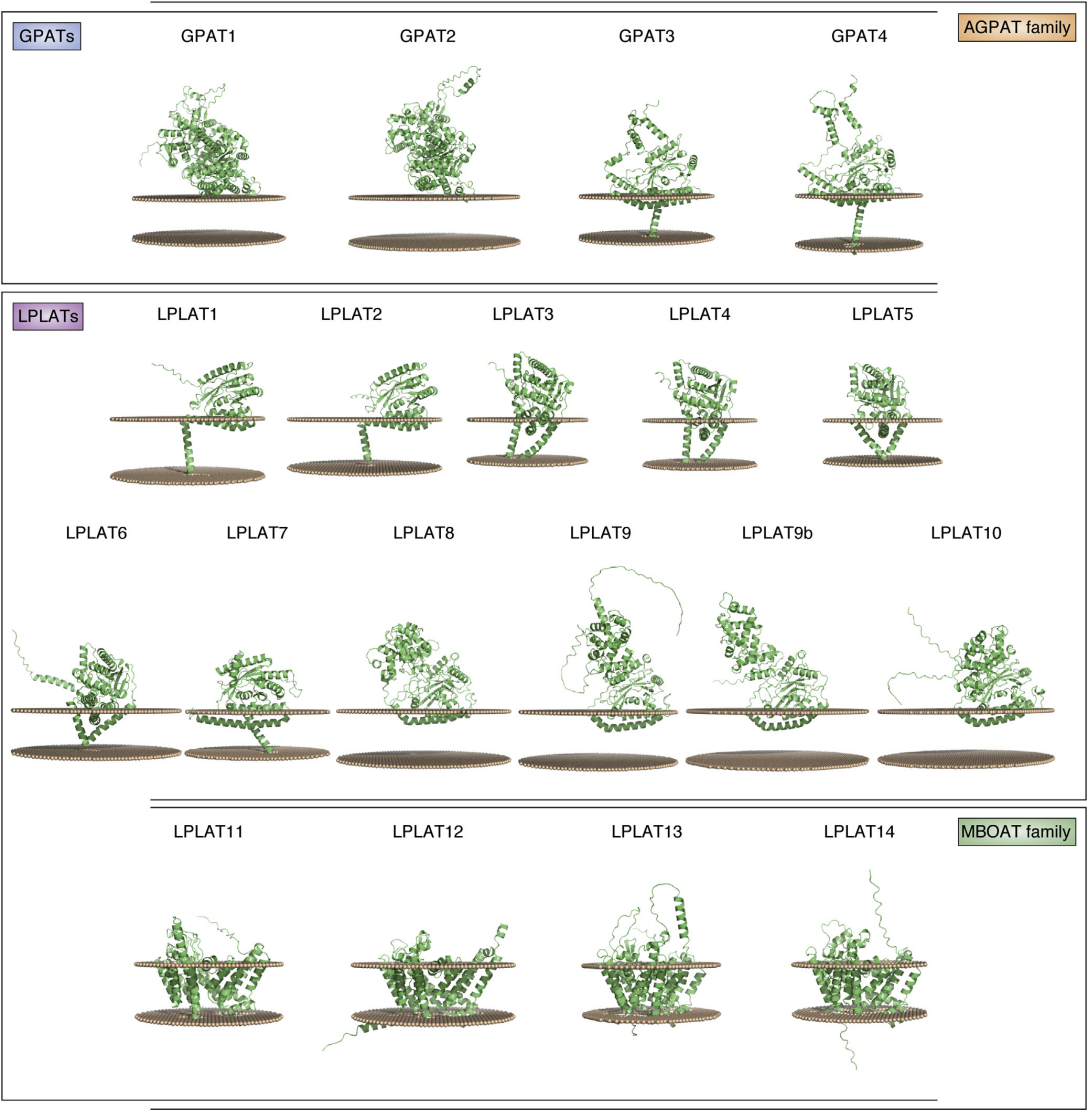
**Membrane-interacting models of AlphaFold structures of human GPATs and LPLATs.** All structures are of human enzymes except for mouse LPLAT9b, which has no human ortholog. Membrane-interacting models were obtained by positioning each AlphaFold structure in a lipid bilayer using the PPM 3.0 Web server, with the option of planar membrane. For each enzyme, two arrays of small spheres represent opposite surfaces of a lipid membrane bilayer. The numbered amino acid residues of regions predicted to be embedded in the membrane are shown in Table 3.

**Figure 8:**
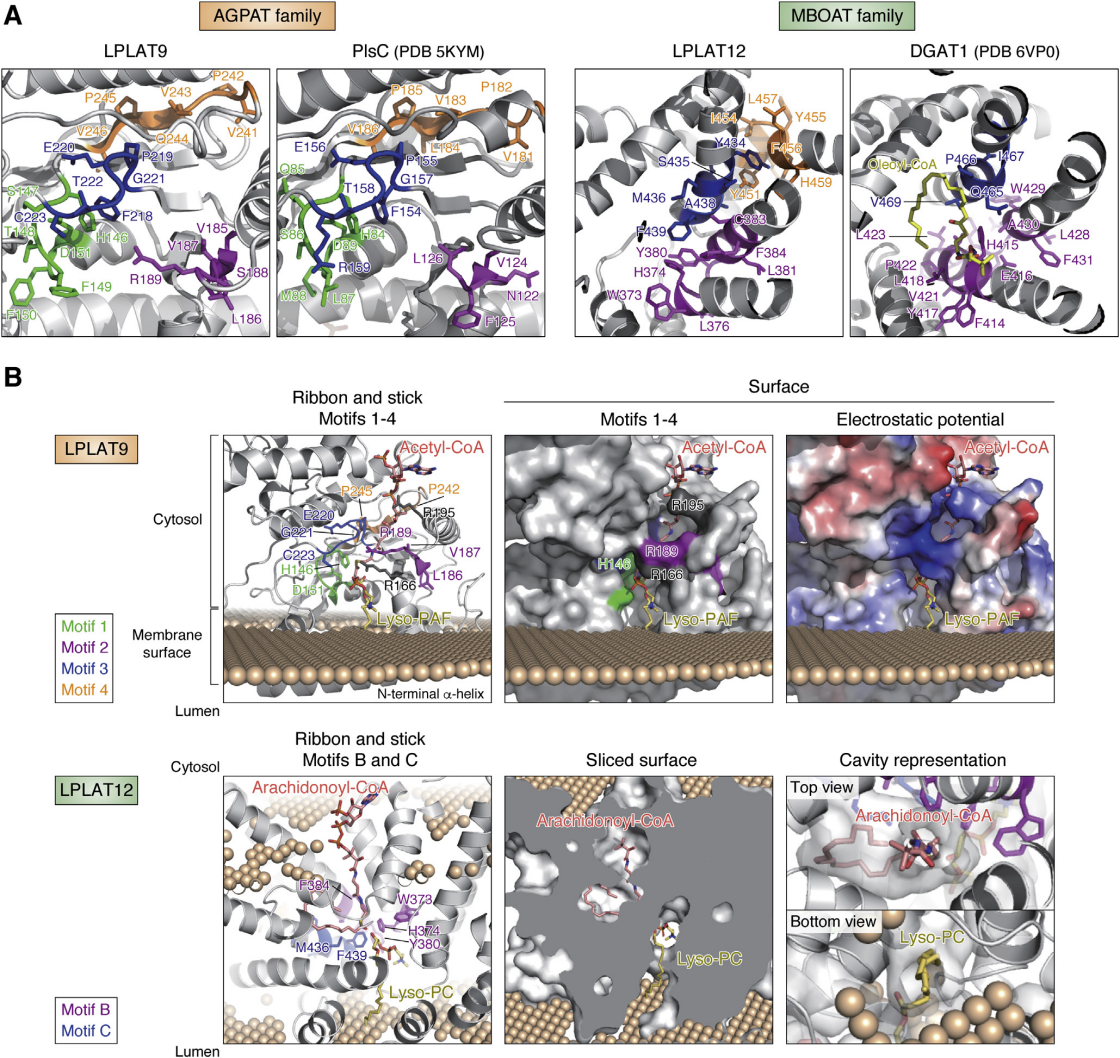
**Magnified views of both motifs and membrane-embedded models of LPLAT9 and LPLAT12.***A*, magnified views of AGPAT Motifs 1 to 4 in LPLAT9 and PlsC (PDB 5KYM) (28) and MBOAT Motifs B to D in LPLAT12 and DGAT1 (PDB 6VP0) (42). For LPLAT9 and PlsC, AGPAT Motifs 1 to 4 are colored *green*, *magenta*, *blue*, and *orange*, respectively. For LPLAT12, MBOAT Motifs B to D are colored *magenta*, *blue*, and *orange*, respectively. For DGAT1, C18:1-CoA is shown in *yellow* bound to the model, with Motifs B and C of DGAT1 colored *magenta* and *blue*, respectively. *B*, membrane-embedded model of LPLAT9 and LPLAT12 was prepared as in Figure 7. Acetyl-CoA and lyso-PAF were manually incorporated into LPLAT9, and arachidonoyl-CoA and lyso-PC were manually incorporated into LPLAT12. Surface model represents the protein surface for LPLAT9 and the cavity surface for LPLAT12. Array of spheres represents membrane surface. Note that the membrane orientation of the LPLAT12, with arachidonoyl-CoA entering from the cytosol and lyso-PC entering from the luminal side of the membrane, is opposite of a recently proposed model by Zhang et al. (43) (discussed more in main text).

Recently, a computational machine learning method named AlphaFold has been developed that can predict protein structures with high accuracy, even if no similar experimentally solved structure is available (39). AlphaFold was utilized to generate structural predictions of almost all human proteins, including LPLATs and GPATs (40). Figure 5, Figure 6, Figure 7, Figure 8 summarize the AlphaFold-generated structural predictions of GPATs and LPLATs, which were predicted with high confidence in most regions of each enzyme (Fig. 5). The predicted structures for all AGPAT-family LPLATs show conserved enzymatic core structures that contain Motifs 1 to 4 (Fig. 5 and Table 3), similar to PlsC (28). In addition, these predicted structures reveal unique regions in each structure, some of which could function as transmembrane helices (Fig. 6). Positioning of the AlphaFold structures in lipid bilayer membranes by computational modeling using PPM 3.0 Web Server (41) suggests that LPLAT8 to 10 are anchored to the membrane by N-terminal helices embedded in one leaflet of the membrane, similarly as reported for PlsC (28). In contrast, LPLAT1 to 7 and GPAT3 to 4 are predicted to more strongly anchor to membranes *via* one or two transmembrane helices, while GPAT1 to 2 are predicted to interact weakly with the membrane surface (Fig. 7 and Table 3). Similar positioning of the AlphaFold structures for the MBOAT-family LPLATs, LPLAT11 to 14, indicated that each of these enzymes may possess 11 transmembrane helices (Fig. 7 and Table 3), comparable to the experimentally determined structures of non-LPLAT MBOAT family members such as human HHAT (12 transmembrane helices) (32) or human DGAT1 (nine transmembrane helices) (31, 42).

As representative LPLATs of the AGPAT and MBOAT families, detailed views of the AGPAT Motifs 1 to 4 in LPLAT9 (LPCAT2) and MBOAT Motifs B-D in LPLAT12 (LPCAT3) are shown in Figure 8*A*. These motifs are clustered to form a cavity where acyl-CoAs and lysophospholipids might bind, as also shown in the solved structures for PlsC (28) and DGAT1 (42) (right panels). Figure 8*B*, upper panel, shows a manually docked model of LPLAT9 with acetyl-CoA and lyso-platelet-activating factor (lyso-PAF), the natural substrates utilized by LPLAT9 in PAF production. LPLAT9 possesses a hydrophobic cavity that faces the cytosol at one end and the membrane surface at the other, and acetyl-CoA and lyso-PAF could enter the cavity from the cytosol and the membrane, respectively, contacting with each other in the vicinity of the catalytic His146. AGPAT Motifs 1 to 3 constitute the cavity, whereas Motif 4 functions as a backing for the cavity. Besides the motifs, Arg166 and Arg195 could function in recognizing the phosphate groups in lyso-PAF and acetyl-CoA, respectively. Figure 8*B*, lower panel, shows a manually docked model of LPLAT12 with arachidonoyl-CoA and lyso-PC, the natural substrates utilized by LPLAT12 in arachidonoyl-containing-PC production. LPLAT12 possesses a hydrophobic cavity at the center that penetrates the membrane, and arachidonoyl-CoA and lyso-PC could bind to the cavity from the cytosol and the luminal side of the membrane, respectively, contacting with each other in the vicinity of the catalytic His374. MBOAT Motifs B and C, but not A or D, contribute to the construction of the cavity, which possesses a hydrophobic bulge at the center that might be suitable for accommodating the arachidonoyl chain. Recently, Zhang et al. reported on the structure of the LPLAT12 ortholog in chicken as determined by x-ray crystallography, cryo-EM, and sequence analysis (43). The topology of their model with an ER lumen-facing gate for acyl-CoA substrate was surprising in being oriented opposite of to other determined structures for the MBOAT proteins SOAT1, DGAT1, and HHAT (29, 30, 31, 32, 42, 44). The lumen-/cytosol-facing orientations of their model is also opposite to that presented in our modeled structural prediction, and more analyses are needed to firmly establish the topology and membrane orientation of LPLAT12.

Future experimental studies will be required to validate the predicted structures of LPLATs in both families and reveal their true forms, membrane topologies, and catalytic mechanisms. It is expected that the current advances in protein structural predictions for GPATs and LPLATs may hold potential to offer insights regarding the structural bases for substrate access, specificity, product release, and inhibitors development for all of the enzymes. Combined with experimental validation, those studies may go far to fill in the gaps that still exist in our knowledge of the biochemical activities and biological functions of each enzyme, which are summarized in the following sections for GPATs (Characteristics of GPATs section) and LPLATs (Characteristics of lysophospholipid acyltransferases section).

## Characteristics of GPATs

GPATs function in the common biosynthetic pathways of *de novo* phospholipid synthesis and TAG production by esterification of a fatty acid at the *sn*-1 or *sn*-2 position of G3P to generate LPA. At present, four GPAT enzymes, GPAT1-4, have been identified (Table 2). All four GPATs belong to the AGPAT family and have AGPAT motifs (18). GPAT1 and 2 are mitochondrial GPATs and localized on the outer mitochondrial membranes, while GPAT3 and 4 are microsomal GPATs and localized in the endoplasmic reticulum (ER) (18, 45). In this section, we briefly summarize the biochemical and biological characteristics of GPATs.

### GPAT1

The mitochondrial enzyme GPAT1 (also called GPAM) is highly expressed in lipogenic tissues such as liver and adipose, where its levels are decreased by fasting and increased by insulin, indicating a metabolic function to regulate fat utilization and storage (46, 47). GPAT1 is resistant to N-ethylmaleimide (NEM)-induced inhibition, unlike the other three GPATs (48). GPAT1 has selectivity to incorporate saturated acyl-CoAs such as C16:0 as substrates (46, 47, 49), and GPAT1-knockout (KO) mice had phospholipid alterations that included reduced C16:0 in *sn*-1 of PC and PE and increased C20:4 in *sn*-2, indicating that GPAT1 may not only function in TAG production but also influence the fatty acid compositions of phospholipids (50, 51). In most tissues, GPAT1 only accounts for a minority of GPAT activity (∼10%), but is abundant in the liver where it is suggested to account for 20 to 50% of all GPAT activity (48, 52).

### GPAT2

GPAT2 (also called xGPAT1) shows selectivity to utilize C20:4-CoA as substrate *in vitro* assays (53). Among several tissues in mice, GPAT2 mRNA was expressed almost exclusively in testis, where it was detected in germ cells. The expression in the liver was at least 50-fold less, and even lower in other tissues including the brown adipose tissue (BAT), brain, lung, and heart (53). This expression pattern suggests GPAT2 may have a primary function in the testis but not in TAG production in most tissues (54, 55). GPAT2 is also highly expressed in tumors and was suggested to belong to a class of “cancer-testis” genes, whose expression is normally low in somatic tissues but may be upregulated in cancers of various origins (56).

### GPAT3

GPAT3 (also called AGPAT8, AGPAT9, and AGPAT10) is a microsomal GPAT, but was also suggested to possess LPAAT activities (57). During differentiation of 3T3-L1 adipocytes, GPAT3 was highly upregulated and promoted TAG storage in lipid droplets, demonstrating that GPAT3 has a major role in adipogenesis (58, 59). GPAT3-KO mice were reported to have ∼80% reduction of GPAT activity in white adipose tissue (WAT) (60). GPAT3-KO mice had increased energy expenditures on high-fat diet, and females but not males showed decreased adiposity and body weight gain, indicating important roles of GPAT3 in energy and lipid homeostasis (60).

### GPAT4

GPAT4 (also called AGPAT6) is abundantly expressed in various tissues. Its expression is high in the liver, BAT, and WAT, where GPAT4 was reported to be responsible for most GPAT activity (61). GPAT4-KO mice had reduced body weights and were resistant to both diet- and genetically-induced obesity (61). GPAT4-KO mice showed increased energy expenditure, reduced TAG accumulation in BAT and WAT, and profound lack of subdermal adipose tissues. There was an overall increase in polyunsaturated and decrease of monounsaturated fatty acid chains in TAGs, DAGs, and phospholipids; suggesting GPAT4 has selectivity to incorporate monounsaturated fatty acid substrates (61). During lactation, GPAT4 is upregulated in mammary gland epithelia and participates in TAG and DAG synthesis required to support milk production. Indeed, GPAT4-KO mice had dramatically decreased fat droplets within mammary epithelial cells and ducts. GPAT4-KO nursing females had greatly reduced TAG and DAGs in their milk and were unable to successfully nurse their young, indicating a critical role of GPAT4 for the production of milk fat (62).

Recently, calcineurin B homologous protein 1 (CHP1) has been found to be an essential cofactor for GPAT4 and thereby regulate ER glycerolipid synthesis. CHP1 binding was required for GPAT4 activation, and myristoylation of CHP1 was required for the full interaction. Loss of CHP1 in mammalian cells led to severely reduced fatty acid incorporation and storage, but was partially compensated for by upregulation of glyceronephosphate *O*-acyltransferase (GNPAT) and increased synthesis of peroxisomal ether lipids (63).

## Characteristics of lysophospholipid acyltransferases

In this section, biochemical and biological characteristics of each LPLAT are summarized. The new proposed names for LPLATs are also introduced, which are in the format LPLATx, where x is a number (Table 1). As described in Nomenclature section, these names are unambiguous and do not imply substrate specificities. The official gene name is given in parentheses immediately following the first instances of the new names. Enzymatic characteristics for the LPLATs are summarized in Table 4 and Figure 4. Physiological roles of LPLATs and their disease associations are summarized in Table 5.

**Table 4:** Characteristics of LPLATs

Family	LPLAT	Tissue distribution	Subcellular localization^a^	Amino acid number		Type of remodeling^b^	References
				Human	Mouse		
AGPAT	LPLAT1	ubiquitous	ER	283	285		(66, 67, 80, 184)
	LPLAT2	adipose tissue, pancreas, liver, kidney, gut, skeletal muscle	ER	278	278		(67, 80, 186)
	LPLAT3	retina, testis, brain, heart, liver, kidney	ER, Golgi, NE	376	376		(80, 81, 83, 84, 94, 189, 192)
	LPLAT4	brain, lung, stomach, spleen, intestine, colon, testis, gut, ubiquitous	ER	378	378		(80, 88, 90)
	LPLAT5	brain, heart, skeletal muscle, ubiquitous	ER, Mit, NE	364	365		(80, 94)
	LPLAT6	heart, liver, kidney, skeletal muscle, pancreas, spleen	ER	414	376	Type 1	(98, 99, 102, 182, 187)
	LPLAT7	peripheral blood, liver, lung, placenta, kidney, brain	ER	370	370		(115)
	LPLAT8	lung, spleen	ER, LD	534	534	Type 3	(6, 119, 122, 133, 182, 188)
	LPLAT9	macrophages, microglia, neutrophils, spleen, skin	ER, LD	544	544		(128, 129, 133, 135)
	LPLAT9b				516		
	LPLAT10	brain	ER	524	524		(139, 140)
MBOAT	LPLAT11	brain, liver, testis, lung	ER	472	473		(145, 193)
	LPLAT12	liver, testis, small intestine, kidney	ER	487	487	Type 2	(158, 161, 162, 164, 182)
	LPLAT13	epididymis, testis, brain, ovary	ER	520	519		(158)
	LPLAT14	stomach, colon, epididymis	ER	495	492		(158)

a ER, endoplasmic reticulum, Mit, mitochondrial; NE, nuclear envelope; LD, lipid droplet.

b Type 1, *sn*-1 remodeling; Type 2, *sn*-2 remodeling; Type 3, *sn*-1/2 remodeling.

**Table 5:** Physiological roles of LPLATs and disease associations

Proposed name	Phenotype and lipid alterations in gene-deficient mice	Human disease associations
LPLAT1/AGPAT1	nervous and reproductive system abnormalities, impaired fat storage and lipid homeostasis (69)	allelic risk association for exfoliation syndrome (70)
LPLAT2/AGPAT2	lipodystrophy (76)	mutational loss causes lipodystrophy (75); up-regulated in gynecological cancers (71, 72, 73)
LPLAT3/AGPAT3	visual dysfunction, male infertility, hepatic PUFA level control, major reduction in DHA-containing phospholipid (83, 84, 85)	
LPLAT4/AGPAT4	learning and memory deficits (89, 91); multiple lipid alterations and compensatory upregulation of other LPLATs (89, 92, 93)	
LPLAT5/AGPAT5	insulin-resistance (knockdown model) (97)	
LPLAT6/LCLAT1	altered PIP signaling, resistant to mitochondrial and oxidative-stress-related diseases including obesity, insulin resistance (101), steatosis (105), Parkinson's disease (108), and others (106, 107); exacerbated fibrosis in IPF model (knockdown model) (112); major reduction of C18:0 chains in PI, PIPs, and PIP_2_s (102, 103)	increased in idiopathic pulmonary fibrosis (IPF) patients' lung tissues and is candidate genetic variant susceptibility gene for IPF (112); up-regulated in lung tumors (111)
LPLAT7/LPGAT1	resist obesity but prone to insulin resistance and severe hepatosteatosis on high-fat diet (116, 117); altered acyl chain compositions of several phospholipid classes (116)	obesity-related trait in Pima Indians (118)
LPLAT8/LPCAT1	decreased lung surfactant and function (6, 122), retinal degeneration (123)major reduction in C16:0-containing PC including DPPC (6, 123, 124)	amplified in over half of human cancers (125, 126, 127)
LPLAT9/LPCAT2	decreased neuropathic pain (129); lack of production of inflammatory PAF (129)	possible biomarker or therapeutic target for human allergic diseases (138)
LPLAT10/LPCAT4		
LPLAT11/MBOAT7	pups small, at low frequency and low survival, with abnormal brain development (145, 146); hepatic steatosis induced by hepatic gene silencing (156, 157) or deletion (153, 154, 155); decreased C20:4-containing PI and PI phosphates (145, 146, 153, 154, 155)	mutational loss is associated with intellectual disability, autism and epilepsy (147); key genetic modifier of and possible therapeutic target for fatty liver diseases (148, 150, 152)
LPLAT12/LPCAT3	neonatal lethality, nutritional failure, variable TAG accumulation in intestines (162, 163, 164), intestinal tumorigenesis (167); deletion in nervous system caused brain microgliosis and auditory dysfunction (174); deletion in skeletal muscle protects from HFD-induced insulin resistance (175); acute deletion in small intestine reduces lipid uptake and reduces lipid and lipoprotein levels in plasma (165); major reduction in C18:2 and C20:4-containing phospholipid (162, 164, 165, 175)	
LPLAT13/MBOAT2		
LPLAT14/MBOAT1		disrupted by translocation in a patient with novel brachydactyly-syndactyly syndrome (178); lies in multi-gene deletion region associated with mesomelic dysplasia (179, 180)

### LPLAT1 (AGPAT1)

LPLAT1 (AGPAT1), also sometimes called LPAAT1, utilizes a variety of acyl-CoAs to produce PA during *de novo* phospholipid synthesis as well as TAG production (64, 65, 66, 67) (Table 4 and Fig. 4). LPLAT1 is ubiquitously expressed and functions in several organ systems (68, 69). LPLAT1-KO mice were born at less than Mendelian frequency and had widespread disturbances in metabolism as evidenced by decreased leptin and plasma glucose, reduced total body fat, and reduced epididymal fat pads in males. LPLAT1-KO mice that survived to adulthood had abnormalities in sperm development and ovulation (69). LPLAT1-KO mice also showed reduced hippocampal CA-region thickness, reduced neuron number, and impaired neurological functions as well as features of seizures/epilepsy. Thus, LPLAT1 has essential functions in the central nervous system, the reproductive system, and in maintaining lipid homeostasis (Table 5) (69). A recent genome-wide association study analysis identified a strong association between genetic locus of LPLAT1 and increased risk of exfoliation syndrome, a major cause of blindness (70), suggesting a possible role in this disease that is yet to be elucidated (Table 5).

### LPLAT2 (AGPAT2)

LPLAT2 (AGPAT2), also called LPAAT2, shares high homology with LPLAT1 and has similar biochemical properties to utilize a variety of acyl-CoAs to produce PA for phospholipid as well as TAG biosynthesis (64, 65, 66, 67) (Table 4 and Fig. 4). LPLAT2 is expressed in several tissues including the adipose tissue, pancreas, and liver (67). Elevated expression of LPLAT2 was observed in several types of human cancers, and high expression in endometrial and ovarian cancers was associated with high cancer aggressiveness and poor survival (71, 72, 73). These observations suggested that LPLAT2 inhibition may be a therapeutically promising strategy to treat cancers, which was later also supported by a study utilizing a mouse xenograft model of osteosarcoma (74).

LPLAT2 has an essential physiological role in adipose tissues, where it functions in TAG production and accumulation (68). In humans, LPLAT2 mutations cause type 1 congenital generalized lipodystrophy (CGL, also called Berardinelli–Seip lipodystrophy), a rare disorder that includes a total lack of subcutaneous body fat from birth, increased appetite, accelerated growth, and other metabolic complications including severe insulin resistance, hypertriglyceridemia, hepatic steatosis, and early onset diabetes (75). Similarly, LPLAT2-KO mice have decreased body fat and develop extreme insulin resistance, diabetes, and hepatic steatosis (Table 5) (76). It has been proposed that LPLAT2 mutations cause CGL by inhibiting TAG biosynthesis and storage in adipocytes, which promotes TAG accumulations in other tissues such as the skeletal muscle and liver and causes further imbalances in metabolic homeostasis (76, 77, 78, 79) (Table 5).

### LPLAT3 (AGPAT3)

LPLAT3 (AGPAT3), also sometimes called LPAAT3, utilizes several acyl-CoAs including docosahexaenoic acid (C22:6, DHA)-CoA as donor substrates in *in vitro* assays (80, 81, 82) (Fig. 4). However, analyses using LPLAT3-KO mice showed that LPLAT3 mainly produces DHA-containing phospholipids *in vivo* (83, 84, 85) (Table 5). DHA is essential for visual, reproductive, and brain functions and is thought to have beneficial roles in many other organs that promote improved metabolism. LPLAT3 incorporates DHA into LPA to produce PA, which determines the level of DHA-containing phospholipids in many tissues including the sperm, retina, muscle, and liver (83, 84, 85, 86) (Table 4 and Fig. 4).

Enrichment of polyunsaturated fatty acids such as DHA is predicted to impart flexibility to biological membranes (9, 11), and LPLAT3-KO mice have been used to elucidate the physiological effects of DHA-containing phospholipids. The mice showed at least three abnormalities, which indicate essential roles for DHA-containing phospholipids *in vivo*. LPLAT3-KO male mice are infertile and their sperm possess an excess of cytoplasm from Sertoli cells around the sperm heads. DHA-containing phospholipids may be required for rapid endocytosis and clearance of excess cytoplasm through narrow tubules at sperm–Sertoli cell contacts during late sperm maturation (84). Disordered retinal disc morphology in LPLAT3-KO mice also revealed the essential requirement of DHA-containing phospholipids for photoreceptor cell disc formation and maintenance (83). Liver has membranes rich in DHA (6); however, its biological significance has not been elucidated. Notably, reduction of hepatic DHA-containing phospholipid by global as well as hepatocyte-specific deletion of LPLAT3 leads to a compensatory increase of arachidonic-acid-containing phospholipids not only in the liver but also in other tissues including the brain. Therefore, it is proposed that hepatic DHA-containing phospholipids work as the molecular rheostat for systemic homeostasis of systemic polyunsaturated fatty acid levels (85). Polyunsaturated fatty acid–containing phospholipids produced by LPLAT3 may have other roles. For example, a recent genome-wide CRISPR-Cas9 suppressor screening has revealed that LPLAT3-dependent production of polyunsaturated fatty acid–containing ether lipids is essential for susceptibility to ferroptotic cell death (87).

### LPLAT4 (AGPAT4)

LPLAT4 (AGPAT4), also called LPAAT4, is expressed in many tissues including the brain, muscle, and WAT and has activity to incorporate unsaturated fatty acids into LPA to produce PA; with selectivity to utilize C22:6->C20:4->C18:2->C18:1->C16:0-CoAs as donor substrates (80, 88) (Table 4 and Fig. 4). LPLAT4 shows a unique organelle distribution. It is localized in outer mitochondrial membranes in specific brain regions (88, 89). LPLAT4 also preferentially localizes in the *trans*-Golgi and *trans*-Golgi network, where it produces PA, which is essential for Golgi membrane fission (90). Brains from LPLAT4-KO mice showed compensatory increase in several other LPLATs but decreased levels of PC, PE, and PI. This suggests that LPLAT4 has a role to produce PA that is precursor for PI, PC, and PE in brain (89). LPLAT4-KO mice had deficits in learning and memory (Table 5). This was associated with reduced expression of glutamate receptors, although underlying mechanisms remain unclear (91). LPLAT4 -KO mice also showed selective compensatory gene expression patterns in WAT tissues, with several isoforms of LPLATs (LPLAT1, 2, 3, and 5) and GPAT1-4 upregulated in perirenal but not epididymal WAT; while, interestingly, TAG and PA levels were increased in epididymal but not in perirenal WAT (92). In skeletal muscle, LPLAT4 levels were significantly higher in oxidative soleus muscle than in extensor digitorum longus or gastrocnemius (93). LPLAT4-KO mice showed compensatory upregulation of LPLAT2 and LPLAT5 (AGPAT5) in soleus, along with increased levels of PC and PE (93). The studies with LPLAT4-KO mice have indicated roles for LPLAT4 in several highly metabolically active tissues (*i.e.*, brain, soleus, WAT), which may be partly compensated for by upregulation of other AGPATs in LPLAT4-KO tissues. Further analyses are required to understand the unique biological functions of LPLAT4.

### LPLAT5 (AGPAT5)

*In vitro* studies using cells overexpressing LPLAT5 (AGPAT5) revealed that it colocalized to mitochondria and showed both LPAAT and LPEAT activities with C18:1-CoA (80, 94) (Table 4 and Fig. 4). Gene linkage analyses indicated LPLAT5 gene polymorphisms influence skeletal muscle fat deposition and/or metabolism in pigs and cows (95, 96), as well as being a genetic factor contributing to insulin resistance in a study of over 100 strains of mice (97). Systemic LPLAT5 silencing by antisense oligos improved insulin sensitivity in mice and rats after challenge with high-fat or high-glycemic diets (97) (Table 5), warranting further investigations. Future studies using KO mice may aid in clarifying the physiological functions and activities of LPLAT5.

### LPLAT6 (LCLAT1)

LPLAT6 (LCLAT1), also sometimes called LYCAT, ALCAT1, or AGPAT8, acylates diverse substrates including lyso-PI (LPI), lyso-PG (LPG), and LCL using a variety of acyl-CoAs (98, 99, 100) (Table 4 and Fig. 4). LPLAT6 was initially reported to regulate fatty acid remodeling of CL (98, 101). Later studies indicated that LPLAT6 deletion in mice resulted in altered acyl chain compositions that were most pronounced in PI; the changes were also reflected in PI-derived signaling molecules including PIPs and PIP_2_s. LPLAT6-KO mice had highly decreased activities to incorporate C18:0 into *sn*-2-acyl-LPI, and highly decreased C18:0 chains in PI in various tissues (102). Indeed, PI can be a precursor for phosphoinositides, and in liver the altered fatty acid compositions of PI were preserved in the fatty acid compositions of PIPs and PIP_2_s (102). It has been also reported that LPLAT6 controls the amount and fatty acid composition of specific phosphoinositides and related membrane traffic (103) (Table 5). Homologues in *Caenorhabditis elegans* show similar biochemical and *in vivo* activities to incorporate C18:0 into the *sn*-1 position of *sn*-2-acyl-LPI, indicating that the biological role of LPLAT6 to regulate PI molecular species is evolutionarily conserved from worms to mammals (99, 102).

Key roles of LPLAT6 linking oxidative stress and mitochondrial dysfunction through CL remodeling have been proposed by various studies. Mouse embryonic fibroblasts from LPLAT6-KO embryos are protected from oxidative-stress-induced mitochondrial fragmentation (104). LPLAT6-KO mice show prevention from the onset and progression of various mitochondrial dysfunction-related pathologies, including diet-induced obesity and insulin resistance (101), nonalcoholic fatty liver diseases (105), hypertrophic cardiomyopathy (106), renal injury by myocardial infarction (107), and Parkinson’s disease (108) (Table 5).

LPLAT6 is also has other biological functions. LPLAT6 is reported to control development and fate of hematopoietic and endothelial stem cell lineages, which was observed during *in vitro* differentiation of mouse embryonic stem cells (109). Similarly, in zebrafish, *in vivo* deletion or reduction of the LPLAT6 ortholog resulted in decreases of both endothelial and hematopoietic lineages (110). LPLAT6 is also proposed to regulate fatty acid compositions of mitochondrial CL and to modulate mitochondrial dynamics, with possible roles in nonsquamous cell lung carcinoma progression (111). A role for LPLAT6 is also proposed in idiopathic pulmonary fibrosis (IPF), where LPLAT6 levels were increased in IPF patients' lung tissues (112). In murine IPF models, LPLAT6 overexpression reduced lung fibrosis, while conversely LPLAT6 downregulation increased fibrogenesis. Potential mechanisms of LPLAT6-mediated lung protection were suggested to involve modulation of mitochondrial reactive oxygen species generation and apoptosis in alveolar epithelial cells, raising the possibility that modulation of LPLAT6 activity may offer a new therapeutic approach to improve or slow progression of IPF (112) (Table 5).

In *C. elegans*, there are three homologues of LPLAT6, which seem to have redundant functions. Triple mutant of the three *C. elegans* LPLAT6 homologues, acl-8, -9, and -10, showed abnormalities in the arrangement of the nematode epidermal cells, called seam cells (113). In this mutant, perturbation of PI metabolism was detected. Interestingly, a mutant of an intracellular phospholipase A_1_ (ipla-1) also showed similar abnormalities in seam cells (114), suggesting that they may function in the same axis. Thus, fatty acid remodeling, in which fatty acids at the *sn*-1 position of PI are hydrolyzed by ipla-1 and fatty acid introduction by acl-8, -9, and -10, may determine the arrangement of seam cells. The role of LPLAT6 in the *sn*-1 position remodeling is further discussed in Positional specificities of LPLATs to introduce fatty acids section.

### LPLAT7 (LPGAT1)

When overexpressed in mammalian cell lines, LPLAT7 (LPG acyltransferase [LPGAT]1) localized to ER and had activity to incorporate C16:0, C18:0, and C18:1 into LPG (115) (Table 4 and Fig. 4). Livers of LPLAT7-KO mice had significant alterations in acyl chain compositions not just in PG and in CL, produced from PG, but also in other phospholipid classes including PC, PE, PS, PA, and PI (116) (Table 5). LPLAT7-KO mice had reduced body weights, and they were protected from high-fat-diet-induced obesity. However, LPLAT7-KO mice were prone to hepatopathy, insulin resistance, and hepatic steatosis that was mechanistically linked to oxidative stress and mitochondrial dysfunction in hepatocytes (116). In another study, shRNA-mediated knockdown of LPLAT7 in livers of leptin-receptor-defective *db/db* obese mice led to decreased monoacylglycerol acyltransferase (MGAT) and LPGAT activities in mouse livers as well as decreased serum TAG and cholesterol levels, suggesting that LPLAT7 may function as an MGAT and play a significant role in hepatic TAG synthesis and secretion in *db/db* mice (117). However, the metabolic functions of LPGAT1 still remain unclear, as do the complex alternations of acyl chain compositions in multiple phospholipid classes observed in LPLAT7-KO mice (116, 117). Polymorphisms of the LPLAT7 gene promoter were also strongly associated with susceptibility to obesity in Pima Indians (118) (Table 5), and further studies are required to elucidate the biochemical activities and physiological functions of LPLAT7 that impact susceptibilities to obesity, insulin resistance, and hepatic steatosis.

### LPLAT8 (LPCAT1)

LPLAT8 (LPCAT1), also called AGPAT9, AGPAT10, or AYTL2, is expressed in the lung, retina, and other tissues where it generates C16:0-containing PC (119, 120, 121) (Table 4 and Fig. 4). In lung, LPLAT8 is highly expressed in type II alveolar cells (119, 120, 121). LPLAT8-deficient mice generated by two different groups have confirmed that LPLAT8 is a major producer of dipalmitoyl-PC (DPPC) found in pulmonary surfactant, and both mouse strains showed varying degrees of respiratory dysfunction (6, 122) (Table 5).

A natural mutation in LPLAT8 in *rd11* mice causes rod and cone cell loss leading to retinal degeneration; and as in lung, DPPC is also reduced in retina in *rd11* and LPLAT8-KO mice (123, 124). Although the mechanism whereby this causes vision loss is unclear, it was proposed that LPLAT8-produced DPPC may protect against polyunsaturated fatty acid–mediated cytotoxicity in retinas (124). LPLAT8 also possesses PAF-producing activity *in vitro* and is proposed to have a physiological role in noninflammatory PAF production (36), which awaits future validation *in vivo*.

LPLAT8 may have a major role in the progression of human cancers (125, 126, 127). It is proposed that LPLAT8-produced saturated PC promotes oncogenic growth factor signaling by providing C16:0-containing-PC-rich membrane domains that support receptor tyrosine kinase signaling, including activating mutations of EGFR such as EGFRvIII, a common driver in glioblastoma. LPLAT8 expression is amplified in nearly half of all cancer cases, and LPLAT8 inhibition may be a promising therapeutic target for a wide variety of oncogenic growth factor–driven cancers (126) (Table 5).

### LPLAT9 (LPCAT2)

LPLAT9 (LPCAT2), also called AGPAT11 or AYTL1, incorporates C2:0 (acetic acid) into lyso-PAF to produce PAF (128, 129) (Fig. 4). LPLAT9 also produces PC *in vitro* (6, 128). The activity of LPLAT9 is notably calcium ion-dependent, and LPLAT9 is expressed in inflammatory cells, such as macrophages and neutrophils (Table 4). LPLAT9 is the only LPLAT reported to be regulated by extracellular stimuli. In macrophages, the activity of LPLAT9 is regulated in three independent phases. (i) LPLAT9 is rapidly activated by its phosphorylation at Ser34 within 30 s of stimulation with PAF or ATP (130). (ii) Ser34 of LPLAT9 is also phosphorylated in response to 30 min of stimulation with lipopolysaccharide (129, 131). (iii) Lipopolysaccharide treatment for 16 h upregulates LPLAT9 mRNA and protein (128, 129). Unlike LPLAT9, LPLAT8 is rather constitutively expressed (128, 130, 131). This relationship is similar to that of cyclooxygenase (COX)-1, which is constitutively active, and COX-2, which is activated in response to inflammation (132).

LPLAT9 is found to be localized in ER (128) and lipid droplets (133) (Table 4) and has a possible role in resistance to chemotherapy in colorectal cancer (134, 135). Lipid droplets are known to contribute to cancer progression, and in colorectal cancer patient samples, LPLAT9 expression positively correlated with lipid droplet accumulation. In colorectal cancer cell lines, LPLAT9 expression supported increased lipid droplet accumulation and imparted resistance to chemotherapeutic drug-induced ER stress and caspase activation *in vitro* and impaired immunogenic anticancer responses *in vivo* (134, 135). Further studies are required to elucidate the roles of LPLAT9 in lipid droplet accumulation associated with tumor resistance to chemotherapy, as well as delineate the precise roles of lipid droplets in cancer development and progression.

As a major enzyme for PAF production, LPLAT9 may play a central role in various pathological conditions where PAF and PAF receptor (PAFR) are involved (136). Neuropathic pain is one such condition, and the role of LPLAT9 was confirmed by using KO mice. LPLAT9-KO mice have nearly normal PC levels but dramatically reduced PAF levels, and they are resistant to nerve injury-induced neuropathic allodynia similar to PAFR-KO mice (129, 137) (Table 5). This indicates the importance of the LPLAT9-PAFR axis in the pathology of neuropathic pain and raises hopes that PAF-pain-loop blockers such as PAFR antagonists or LPLAT9 inhibitors may represent a promising new class of analgesic drugs (129). LPLAT9 is also implicated in IgE over-production in human allergic inflammation, and would be a novel therapeutic target for general allergic diseases, since an epigenome association study has identified LPLAT9 as the least methylated loci associated with high IgE serum concentration (138) (Table 5). A homologue of LPLAT9 is reported in the NCBI database to occur in mice, which is tentatively called LPLAT9b (LPCAT2b). It is currently unknown whether LPLAT9b has LPLAT or other enzymatic activities.

### LPLAT10 (LPCAT4)

LPLAT10 (LPCAT4), also called LPEAT2, has activities to incorporate C16:0-CoA, C18:0-CoA, and C18:1-CoA into LPE, LPC, and LPG as well as alkyl-LPC and alkenyl-LPE, and suggested to have a role for biosynthesis of ethanolamine-containing phospholipids, especially in brain (139) (Table 4 and Fig. 4). LPLAT10 is also reported to have endogenous activity to incorporate C22:6-CoA into LPE and LPC, and shape 22:6/20:4 ratios of phospholipids in a Neuro-2A cell line (140) (Fig. 4). A suggested role for LPLAT10 in osteoclast fusion through the rearrangement of PE was also reported (141).

### LPLAT11 (MBOAT7)

PI is exceptionally rich in arachidonic acid (AA, C20:4) (142, 143), although the biological significance of this enrichment is not well known. In a genetic screen of *C. elegans*, mboa-7 was discovered to selectively incorporate C20:4 into LPI, as does the closest mammalian homologue LPLAT11 (MBOAT7), also called LPI acyltransferase (LPIAT)1 (144) (Table 4 and Fig. 4). LPLAT11-KO mice were born at low frequency and had low birth weights and poor survival (Table 5). The mice also had abnormal brain development that was accompanied by decreased C20:4-containing PI and PI phosphates in the brain (145, 146). In these mice, activity to incorporate C20:4 into LPI was almost abolished in several tissues including the brain, whereas activities to incorporate C20:4 into other lysophospholipids were not affected, indicating the physiological role for LPLAT11 to selectively produce C20:4-containing PI (145). The associated brain developmental defects included delayed neuronal migration, disordered cortical lamination and neuronal processes, and small cerebral cortex and hippocampus (145, 146). In humans, genetic loss of LPLAT11 activity is also associated with intellectual disability, autism, and epilepsy, indicating that the requirement for LPLAT11-generated C20:4-containing PI during brain development is conserved between mice and humans (147) (Table 5).

Recent studies have established another role of LPLAT11 as a key genetic modifier of fatty liver diseases. The LPLAT11 rs641738 variant is associated with fatty liver diseases, apparently involving altered LPLAT11 expression and associated acyl chain compositions of membrane phospholipids (148, 149, 150). Although disparate results among studies indicated that the roles of LPLAT11 in liver disease may vary between populations (151), a recent meta-analysis of more than one million participants has confirmed the variant is a risk factor for presence and severity of nonalcoholic fatty liver disease in Caucasian populations (152) (Table 5).

Recent studies from multiple independent research groups have used hepatocyte-specific LPLAT11-KO mice to assess the molecular mechanisms of how LPLAT11 reduction leads to fat hepatic fat accumulation (153, 154, 155). Similarly, *in vivo* silencing of hepatic LPLAT11 by antisense oligonucleotides induced steatosis in mice (156, 157) (Table 5). Several underlying mechanisms have been proposed for the LPLAT11 depletion-induced hepatic fat accumulation. By combining gene knockout and detailed metabolic flow analysis, Tanaka *et al.* (153) showed that hepatic depletion of LPLAT11 induced a novel noncanonical TAG synthesis pathway in which high PI turnover caused continuous DAG production and fueled increased TAG accumulation. This novel pathway may represent a future therapeutic target to treat nonalcoholic fatty-liver disease.

### LPLAT12 (LPCAT3)

LPLAT12 (LPCAT3), also called MBOAT5, can utilize a variety of substrates and shows selectivity to incorporate C18:2 and C20:4 into LPC, LPE, and LPS in *in vitro* assays (158, 159, 160, 161) (Table 4 and Fig. 4). *In vivo*, LPLAT12 regulates C18:2 and C20:4 incorporation into the membrane during Lands cycle reactions in multiple organs (162).

Much has been known about the biological roles of LPLAT12, primarily from studies of KO mice (Table 5). Global (162, 163) as well as intestine-specific (164) LPLAT12-KO mice showed postnatal growth retardation and lethality, possibly due to intestinal lipid malabsorption and transfer. Adult deletion of intestinal LPLAT12 rapidly caused lipid malabsorption and body weight loss (165). At neonatal stages, massive TAG accumulation in enterocytes suggested that LPLAT12-generated C18:2- and C20:4-rich membranes are important for TAG mobilization and secretion, possibly by supporting successful transport to lipoproteins (162, 164). In addition, LPLAT12 is essential for intestinal lipid absorption by modulating expression of lipid uptake-related genes (163) and regulating intestinal membrane fluidity (165, 166). Furthermore, intestinal LPLAT12 plays a regulatory role for gut hormone production in the gut–brain feedback loop (166).

LPLAT12 also plays an important role in intestinal stem cells. LPLAT12-KO mice had dramatically longer and larger small intestines than control mice, which was accompanied by hyperproliferation of intestinal stem cells (163, 167). As an underlying mechanism, it was proposed that LPLAT12-deficiency increased membrane saturation, which in turn transcriptionally stimulated a sterol regulatory element binding protein (SREBP)-2-dependent cholesterol biosynthesis pathway that promoted intestinal stem cell proliferation. Indeed, in intestinal tumor-prone Apc^Min(+/−)^ mice, LPLAT12-deficiency drastically increased tumor formation *via* disrupted cholesterol homeostasis (167).

LPLAT12 is also highly expressed in the liver and plays essential role in fat mobilization in the liver as in the intestine. Liver-specific deletion (164) or knockdown (168) of LPLAT12 leads to reduced plasma TAG and increased hepatic TAG accumulation following challenge by high-fat diet, partly due to defective lipoprotein secretion into circulation (164). Accordingly, adenovirus-mediated LPLAT12 overexpression reduced postprandial lipid levels in the liver and improved plasma lipoprotein profiles in mice (169). Hepatic LPLAT12 regulates the lipogenic response to feeding by promoting processing of SREBP-1c, a central regulator of lipogenesis. LPLAT12-generated C18:2- and C20:4-rich PCs in ER accelerated SREBP-1c processing during feeding and conditions of obesity (170). Similarly, knockdown of hepatic LPLAT12 in the leptin-deficient *ob/ob* mouse model (genetic obesity model) led to reduced hepatic TAG levels through suppression of lipogenesis, indicating that LPLAT12-regulated phospholipid compositions are important regulators of SREBP-1c activation and lipogenesis (170).

LPLAT12 may also have important roles in macrophages. Three independent studies have analyzed *in vivo* roles of LPLAT12 in macrophages but with variations reported among the results. LPLAT12-deficient macrophages were reported to have reduced cholesterol efflux (171, 172) or upregulation of inflammatory cytokines (173). In one study, mice reconstituted with LPLAT12-deficient hematopoietic cells developed more severe atherosclerotic lesions in the *Ldlr*-KO atherosclerosis model (171), while the other studies did not detect appreciable roles of LPLAT12 in macrophages or hematopoietic cells on atherosclerotic lesion formation in the same genetic atherosclerosis model (172, 173). Exacerbated hepatic steatosis was also observed, not only in liver-specific (164), but also myeloid-specific LPLAT12-KO mice when fed a high-fat diet (172), suggesting that not only hepatic but also myeloid LPLAT12 is involved in liver neutral lipid metabolism.

Recent studies have revealed additional roles of LPLAT12 in other organs such as the brain and skeletal muscle (Table 5). In mice with nervous-system-specific KO of LPLAT12, brain microgliosis and auditory dysfunction were observed, although the precise underlying mechanisms remain unclear (174). Skeletal-muscle-specific LPLAT12-KO mice were protected from high-fat-diet-induced insulin resistance, while skeletal-muscle-specific overexpression of LPLAT12 dampened glucose tolerance (175). Mechanistically, it was proposed that LPLAT12-dependent LPC to PC conversion leads to the disruption of plasma membrane organization, which affects insulin receptor phosphorylation and signaling (175).

### LPLAT13 (MBOAT2)

LPLAT13 (MBOAT2), also called LPCAT4, has both LPCAT and LPEAT activities in biochemical assays and selectivities to incorporate C18:1 into LPC and LPE (158, 159) (Fig. 4). LPLAT13 mRNA is highly expressed in the epididymis, brain, testis, and ovary; however, physiological functions in these tissues are unknown (158) (Table 4). LPLAT13 was upregulated during *in vitro* differentiation of chondrogenic cell lines and suggested to function in transition of chondrocytes into hypertrophic chondrocyte and/or mineralized phenotypes (176).

### LPLAT14 (MBOAT1)

LPLAT14 (MBOAT1), also called LPEAT1, exhibits LPEAT and LPS acyltransferase (LPSAT) activities and has selectivity to utilize C18:1-CoA as an acyl donor substrate *in vitro* assays (158, 159) (Table 4 and Fig. 4). In a neuronal cell line differentiation model, knockdown of LPLAT14 leads to the decrease of LPEAT activity utilizing C16:0-, C18:1, and C20:4-CoA, accompanied by reductions in neurite numbers, neuronal marker expression, and voltage-dependent calcium channel activity, indicating a role for LPLAT14 in neuronal differentiation and functions (177). Human LPLAT14 gene disruption by a breakpoint translocation was found in a patient with a novel brachydactyly-syndactyly syndrome (178) (Table 5). In addition, microdeletions of the chromosomal region 6p22.3 that includes LPLAT14 and several additional genes caused mesomelic dysplasia, which also involves shortened middle limb segments (179, 180). As these translocations and microdeletions involved several genes, the possible contribution of LPLAT14 to these disease syndromes is unclear and awaits future analyses using LPLAT14-KO mice.

## Positional specificities of LPLATs to introduce fatty acids

The fatty acids in phospholipid molecules tend to show a nonsymmetrical distribution between *sn*-1 and *sn*-2 chain positions, with saturated fatty acids such as C16:0 and C18:0 or monounsaturated fatty acids such as C18:1 more frequently bound at the *sn*-1 position; whereas polyunsaturated fatty acids such as C18:2, C20:4, or C22:6 are more frequently bound at the *sn*-2 position. As described above, a number of LPLATs have been identified, and these LPLATs appear to make a concerted contribution to the introduction of fatty acids into the *sn*-1 and *sn*-2 positions. Currently, the contribution of individual LPLATs is not clear. However, since C20:4 is almost exclusively detected at the *sn*-2 position of phospholipid molecules, C20:4-introducing LPLATs, such as LPLAT12 (LPCAT3) and LPLAT11 (MBOAT7), are expected to have positional specificities to incorporate fatty acids into the *sn*-2 position.

Although the stereospecifically numbered *sn*-positional specificities of most LPLATs have been largely unknown, several reports within the past decade have examined the *sn*-positional specificities of a small subset of LPLATs. Imae *et al.* (102) reported that LPLAT6 (LCLAT1) mainly introduced C18:0 into the *sn*-1 position of *sn*-2-acyl LPI, and LPLAT6-KO mouse tissues showed decreased LPLAT activities toward *sn*-2-acyl LPI and had decreased amounts of C18:0-containing-PI. These results indicate LPLAT6 is an *sn*-1-position-remodeling-enzyme. Nematode *acl*-8, *acl*-9, and *acl*-10 (113) and yeast PSI1 (181) are homologues of LPLAT6 and have LPIAT activities toward *sn*-2-acyl-LPI, indicating the systems of *sn*-1 position remodeling by these LPLATs are evolutionarily conserved.

Previously, a bottleneck in determination of the *sn*-1 or *sn*-2 positional specificities of LPLATs had been due to technical challenges in preparing purified *sn*-2-acyl-lysophospholipid substrates, which are required to evaluate LPLAT activities to introduce acyl chains specifically at *sn*-1. However, recently a preparation method for the pure *sn*-2-acyl-lysophospholipids was established, and the positional specificities of several LPLATs were evaluated (182). The study revealed that LPLATs could be classified into three types according to their positional specificity for fatty acid incorporation. Among mammalian LPLATs, LPLAT6 is a *sn*-1 position-selective enzyme (type 1), LPLAT12 is a *sn*-2 position-selective enzyme (type 2), and LPLAT8 (LPCAT1) is an enzyme that has specificity to incorporate fatty acids towards both *sn*-1 and *sn*-2 positions (type 3) (Table 4). In the future, the *sn*-positional specificities of all LPLATs will be revealed.

## Conclusion and perspectives

Here we have summarized the current progress on LPLATs of the AGPAT and MBOAT families. These enzymes function in the Kennedy pathway and the Lands cycle to regulate the fatty chain compositions of membrane phospholipids. Much progress in our knowledge of these enzymes has been made in the past decade. Gene-deficient mice for these enzymes have provided us important insights into mechanisms and biological significance of the generation of membrane phospholipid diversity. Another recent advance is determination of positional *sn*-1 and *sn*-2 specificities of several LPLATs, made possible by technological improvements (182), which will further expand our knowledge on the phospholipid diversity in the future. This review updates our previous review in 2009 and also proposes an updated nomenclature that will better serve the research field.

The current nomenclature of LPLATs is complicated, with several cases of individual enzymes being assigned different names by different groups, and the same name being used for different enzymes in some instances. This overlapping nomenclature for some enzymes has caused confusion in the field, and as more studies come out, the inconsistent and overlapping naming creates even more of an impediment in accessing the expanding body of knowledge. This review puts forward a proposed enzyme nomenclature that is not based on the perceived lysophospholipid substrate specificities. It is hoped that in the near future a similar nomenclature might be agreed upon and broadly adopted that provides clear and specific names for each enzyme.

Based on the current trajectory, the LPLAT field is still on the cusp of greatly expanding the known roles for LPLATs. Phospholipid analyses using gene-deficient mice of all of the enzymes, including inducible- and tissue-specific KOs, are expected to continue to accelerate the discovery of important roles for these enzymes in a variety of biological and physiological processes. These discoveries in parallel with ongoing technological advances in biomembrane analyses will greatly expand our knowledge of how/why membrane phospholipid compositions and diversity can impact human health and diseases.

## Conflict of interest

Department of Lipid Signaling, National Center for Global Health and Medicine is financially supported by 10.13039/501100013170ONO PHARMACEUTICAL CO, LTD.
